# The LRRK2 signaling network converges on a centriolar phospho-Rab10/RILPL1 complex to cause deficits in centrosome cohesion and cell polarization

**DOI:** 10.1242/bio.059468

**Published:** 2022-07-29

**Authors:** Antonio Jesús Lara Ordóñez, Rachel Fasiczka, Belén Fernández, Yahaira Naaldijk, Elena Fdez, Marian Blanca Ramírez, Sébastien Phan, Daniela Boassa, Sabine Hilfiker

**Affiliations:** 1 Department of Molecular Biology, Institute of Parasitology and Biomedicine “López-Neyra”, Consejo Superior de Investigaciones Científicas (CSIC), 18016 Granada, Spain; 2 Department of Anesthesiology and Department of Physiology, Pharmacology and Neuroscience, New Jersey Medical School, Rutgers, The State University of New Jersey, Newark, NJ 07103, USA; 3 Department of Neurosciences and National Center for Microscopy and Imaging Research, University of California San Diego, La Jolla, CA 92093, USA

**Keywords:** LRRK2, Rab GTPase, RILPL1, Vps35, PPM1H, Centrosome

## Abstract

The Parkinson's-disease-associated LRRK2 kinase phosphorylates multiple Rab GTPases including Rab8 and Rab10, which enhances their binding to RILPL1 and RILPL2. The nascent interaction between phospho-Rab10 and RILPL1 blocks ciliogenesis *in vitro* and in the intact brain, and interferes with the cohesion of duplicated centrosomes in dividing cells. We show here that regulators of the LRRK2 signaling pathway including vps35 and PPM1H converge upon causing centrosomal deficits. The cohesion alterations do not require the presence of other LRRK2 kinase substrates including Rab12, Rab35 and Rab43 or the presence of RILPL2. Rather, they depend on the RILPL1-mediated centrosomal accumulation of phosphorylated Rab10. RILPL1 localizes to the subdistal appendage of the mother centriole, followed by recruitment of the LRRK2-phosphorylated Rab proteins to cause the centrosomal defects. The centrosomal alterations impair cell polarization as monitored by scratch wound assays which is reverted by LRRK2 kinase inhibition. These data reveal a common molecular pathway by which enhanced LRRK2 kinase activity impacts upon centrosome-related events to alter the normal biology of a cell.

## Abstract (precis)

**Summary:** The Parkinson's disease LRRK2 signaling pathway converges upon the formation of a complex at the subdistal appendage of the mother centriole which causes centrosomal deficits and impairs appropriate cell polarization.

## INTRODUCTION

Autosomal-dominant mutations in the leucine rich repeat kinase 2 (LRRK2) gene cause familial Parkinson's disease (PD), and coding variants in the same gene can act as risk factors for sporadic PD. Known pathogenic LRRK2 mutations produce a protein with increased kinase activity (Alessi and Sammler, 2018; Kluss et al., 2019), raising the possibility that kinase inhibitors may be useful to treat LRRK2-related PD. Therefore, understanding the downstream effects of enhanced LRRK2 kinase activity is of interest to understand the pathobiology of LRRK2 and to develop assays able to stratify patients who may benefit from LRRK2-related therapeutics.

Mass spectroscopy efforts have revealed a subset of Rab GTPases including Rab8, Rab10, Rab12, Rab35 and Rab43 as the primary endogenous substrates of LRRK2 (Steger et al., 2016, 2017; Thirstrup et al., 2017). LRRK2 phosphorylates these substrates in a conserved region of the switch 2 domain, which leads to impaired interactions with various effector and regulatory proteins (Steger et al., 2016). We have previously shown that this can interfere with the physiological functions of these Rab proteins as key regulators of distinct membrane trafficking events (Rivero-Ríos et al., 2019; Rivero-Ríos et al., 2020). Since the stoichiometry of these phosphorylation events is very low, how such small deficits in Rab functioning may contribute to disease in an autosomal-dominant manner remains unclear. Importantly though, once phosphorylated, the Rab proteins gain the ability to bind to a novel set of effector proteins (Steger et al., 2017), suggesting that these nascent interactions may contribute to the pathobiology of LRRK2 in a dominant fashion.

LRRK2-phosphorylated Rab8 and Rab10 bind with great preference to RILPL1 and RILPL2 (Steger et al., 2017), two poorly characterized proteins reported to regulate ciliary content (Schaub and Stearns, 2013). An important and direct consequence of the LRRK2-mediated phosphorylation of Rab10 is a decrease in primary cilia in various cell types *in vitro* as well as in the intact mouse brain (Steger et al., 2017; Dhekne et al., 2018; Lara Ordóñez et al., 2019; Khan et al., 2021), which may negatively impact upon a signaling pathway to maintain dopaminergic cell health (Dhekne et al., 2018). We previously showed that pathogenic LRRK2 also causes deficits in the cohesion between centrosomes in a manner mediated by phospho-Rab8/10 and RILPL1, including in peripheral cells derived from LRRK2 PD patients (Madero-Pérez et al., 2018; Lara Ordóñez et al., 2019). Interfering with appropriate centrosome cohesion by depletion of proteins critical for this process does not cause cell cycle arrest (Fry et al., 1998; Mayor et al., 2000; Faragher and Fry, 2003; Flanagan et al., 2017), but can lead to deficits in cell polarization (Floriot et al., 2015; Panic et al., 2015). However, the cellular consequences of the LRRK2-mediated centrosomal cohesion deficits remain unclear.

Recent studies have described upstream and downstream regulators of the LRRK2 kinase pathway. A point mutation in vps35, the cargo binding component of the retromer complex, causes autosomal-dominant late-onset familial PD (Vilariño-Güell et al., 2011; Zimprich et al., 2011; Sharma et al., 2012) and potently activates the LRRK2 kinase as assessed by phospho-Rab8 and phospho-Rab10 levels in cells and tissues (Mir et al., 2018). Conversely, the PPM1H phosphatase acts as a downstream regulator to counteract LRRK2 signaling by dephosphorylating Rab8 and Rab10 (Berndsen et al., 2019). Finally, LRRK2 harbors several protein coding variants which modulate risk for sporadic PD potentially mediated by subtle alterations in the LRRK2 kinase activity (Kluss et al., 2019). However, it remains unknown whether modulating the LRRK2 kinase pathway by such distinct means causes centrosomal cohesion deficits in all cases.

Here, we show that distinct modulators of the LRRK2 signaling pathway including vps35 and PPM1H converge upon causing centrosomal cohesion deficits in cultured cells. The pathogenic LRRK2-mediated cohesion deficits are independent of the presence of Rab12, Rab35, Rab43 or RILPL2, but depend on the RILPL1-mediated centrosomal accumulation of phosphorylated Rab10. Correlated light and electron microscopy (CLEM) indicates that RILPL1 directs the phosphorylated Rab proteins to subdistal appendages of the mother centriole, where they may interfere with proper centrosomal function. In cells with an increase in endogenous LRRK2 kinase activity, this is associated with abnormal Golgi positioning and deficits in cell polarization. Therefore, the LRRK2 signaling pathway converges on a centriolar phospho-Rab10/RILPL1 complex to cause deficits in centrosome cohesion and cell polarization.

## RESULTS

### Upstream and downstream regulators of the LRRK2 signaling pathway impact upon centrosomal cohesion in A549 cells

**Fig. 1.**
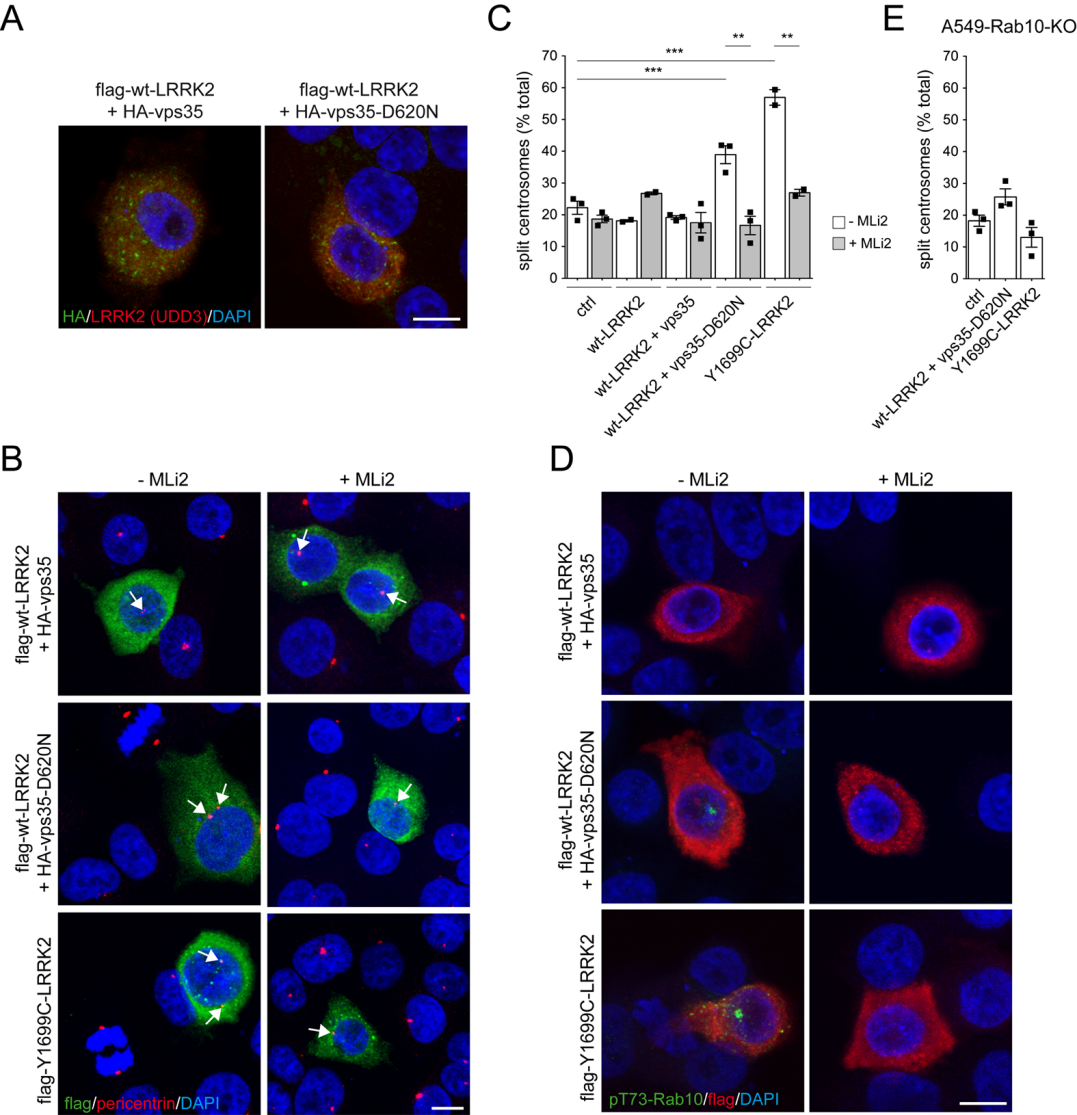
Co-expression of vps35-D620N with wildtype LRRK2 causes centrosomal cohesion deficits dependent on the LRRK2 kinase activity. (A) Example of A549 cells transfected with HA-tagged vps35 or vsp35-D620N and flag-tagged wildtype LRRK2, and stained with an antibody against the HA-tag (green), an antibody against LRRK2 (UDD3) (red) and DAPI (blue). Co-transfection efficiency is >95% in all cases (not shown), and both vps35 and vsp35-D620N display a punctate vesicular staining. Scale bar: 10 µm. (B) Example of A549 cells co-transfected with flag-tagged wildtype LRRK2 and HA-tagged vps35 or vps35-D620N, or transfected with flag-tagged Y1699C LRRK2 in the absence or presence of MLi2 (200 nM, 2 h) as indicated, and stained with antibodies against flag (green), pericentrin (red) and with DAPI. Arrows point to centrosomes in transfected cells. Scale bar: 10 µm. (C) Quantification of the percentage of transfected cells where duplicated centrosomes are >2.5 µm apart (split centrosomes). Bars represent mean±s.e.m. (*n*=3 experiments; *n*=2 for Y1699C-LRRK2); ***P<0.005; **P<0.01. (D) Example of A549 cells transfected with the indicated constructs and treated with or without MLi2 (200 nM, 2 h) before staining with antibodies against phospho-Rab10 (green), flag (red) and with DAPI. Note perinuclear phospho-Rab10 accumulation in cells co-expressing vps35-D620N with wildtype LRRK2 and in cells expressing pathogenic LRRK2. Scale bar: 10 µm. (E) Quantification of the percentage of transfected A549-Rab10 knockout (KO) cells with duplicated split centrosomes. Bars represent mean±s.e.m. (*n*=3 experiments).

Vps35 is a key component of the retromer complex which regulates vesicular trafficking to and from the Golgi complex. Strikingly, a point mutation (vps35-D620N) that causes autosomal-dominant late-onset PD (Vilariño-Güell et al., 2011; Zimprich et al., 2011; Sharma et al., 2012) hyperactivates LRRK2 through a currently unknown mechanism (Mir et al., 2018). We wondered whether vps35 may impact upon the LRRK2-mediated centrosomal cohesion deficits as assessed by measuring the distance between duplicated centrosomes. Pathogenic LRRK2 expression in vps35-deficient A549 cells (Mir et al., 2018) caused centrosomal cohesion deficits identical to those observed in wildtype cells, indicating that the presence of endogenous wildtype vsp35 is not required for this phenotype (Fig. S1). To determine whether the pathogenic vps35-D620N mutant causes centrosomal deficits by activating LRRK2, we coexpressed wildtype flag-tagged LRRK2 with HA-tagged wildtype or mutant vps35 in A549 cells. Both overexpressed wildtype and mutant vps35 displayed a vesicular localization (Fig. 1A). Co-expression of vps35-D620N with wildtype LRRK2 caused a centrosomal cohesion deficit which was reverted by transient application of the LRRK2 kinase inhibitor MLi2, indicating that it was kinase-mediated (Fig. 1B,C).

Highly selective phospho-state-specific antibodies suitable for immunocytochemistry and Western blotting are currently only available against phospho-Rab10 (Lis et al., 2018). Therefore, and even though both phosphorylated Rab8 and Rab10 bind to RILPL1 to trigger the centrosome-related deficits (Steger et al., 2017; Madero-Pérez et al., 2018; Lara Ordóñez et al., 2019), we focused our subsequent analysis on phospho-Rab10. Staining of cells co-expressing vps35-D620N and wildtype LRRK2 revealed the perinuclear accumulation of phospho-Rab10 which was reverted by MLi2 (Fig. 1D). The centrosomal cohesion deficits and the perinuclear accumulation of phospho-Rab10 mediated by vps35-D620N and wildtype LRRK2 were similar to those observed when expressing flag-tagged pathogenic Y1699C-LRRK2 (Fig. 1B-D). In contrast, no effects were detected when co-expressing wildtype vps35 with wildtype LRRK2 (Fig. 1B-D). Centrosomal cohesion deficits were also observed when co-expressing LRRK2 with the vps35-D620N mutant in HEK293T cells, were reverted by MLi2 treatment, and correlated with an increase in the levels of phospho-Rab10, similar to what we observed with pathogenic Y1699C-LRRK2 (Fig. S2). In addition, and as previously described for pathogenic LRRK2 (Lara Ordóñez et al., 2019), the centrosomal cohesion deficits mediated by co-expression of vps35-D620N with wildtype LRRK2 were abolished in cells deficient in Rab10 (Fig. 1E). These data indicate that the vps35-D620N mutant activates the LRRK2 kinase to cause centrosomal deficits in a manner dependent on the phosphorylation of the LRRK2 kinase substrate Rab10.

**Fig. 2.**
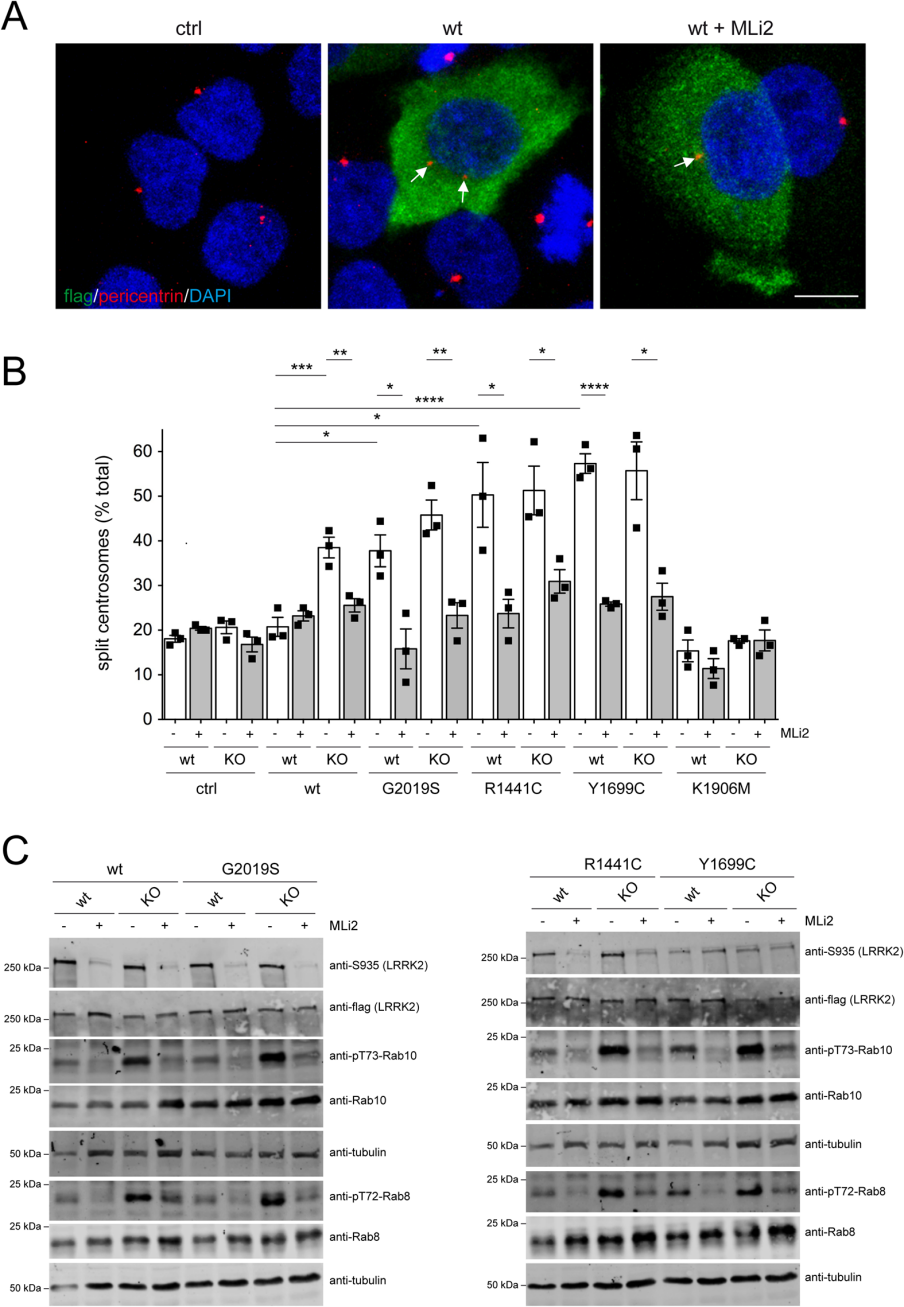
**Wildtype LRRK2 expression causes centrosomal cohesion deficits in A549-PPM1H knockout cells.** (A) Example of PPM1H-KO cells transfected with pCMV (ctrl) or with flag-tagged wildtype LRRK2 in the presence or absence of MLi2 (200 nM, 2 h) as indicated before immunocytochemistry with antibodies against flag (green), pericentrin (red) and with DAPI. Arrows point to centrosomes in transfected cells. Scale bar: 10 µm. (B) Quantification of the percentage of A549 wildtype or PPM1H-KO cells displaying duplicated split centrosomes transfected with either pCMV (ctrl) or the different LRRK2 constructs, and either left untreated or incubated with MLi2 (200 nM, 2 h) prior to immunocytochemistry. Bars represent mean±s.e.m. (*n*=3 experiments); ****P<0.001; ***P<0.005; **P<0.01; *P<0.05. (C) A549 wildtype or PPM1H-KO cells were transfected with the indicated LRRK2 constructs, left untreated, or incubated with MLi2 (200 nM, 2 h) as indicated, and extracts were blotted for flag-tagged LRRK2, phosphorylated LRRK2 (S935), pT73-Rab10, total Rab10, pT72-Rab8a, total Rab8a or tubulin as loading control.

We next evaluated the contribution of PPM1H, the protein phosphatase responsible for dephosphorylating the phosphorylated Rab8 and Rab10 proteins (Berndsen et al., 2019). Side-by-side comparison revealed that wildtype LRRK2 expression *per se* was able to cause a centrosomal cohesion deficit in the PPM1H knockout cells, but not in the wildtype cells (Fig. 2A,B). The centrosomal deficits were reverted by MLi2 and correlated with an increase in the levels of phospho-Rab10 as assessed by immunoblotting (Fig. 2C). No additional effects on centrosomal cohesion were observed when expressing distinct pathogenic LRRK2 mutants in the PPM1H knockout as compared to the control cells (Fig. 2C). Altogether, these data indicate that the centrosomal cohesion deficits mediated by the LRRK2 kinase activity are subject to modulation by both upstream and downstream components of the LRRK2 signaling pathway.

### LRRK2 risk variants modulate centrosomal cohesion in HEK293T cells

To explore the relationship between centrosomal cohesion phenotypes and LRRK2 variants described to positively or negatively impact PD risk, we employed HEK293T cells which express high levels of endogenous Rab10 and display high overexpression levels upon transient transfection, such that subtle changes mediated by risk variants are more likely to be detected. Cells were transfected with wildtype LRRK2, distinct point mutants described to increase PD risk (R1628P, S1647T, N2081D, G2385R), or the pathogenic Y1699C point mutant which served as a positive control (Ross et al., 2008; Lesage et al., 2009; Abdalla-Carvalho et al., 2010; Ross et al., 2011; Zheng et al., 2011; Fu et al., 2013; Hui et al., 2018). Compared to expression of wildtype LRRK2, the pathogenic Y1699C-LRRK2 mutant caused a pronounced deficit in centrosomal cohesion, which was significantly reduced by transient application of the LRRK2 kinase inhibitor MLi2 (Fig. S3A,B), and mild but statistically significant cohesion deficits were also observed when expressing the four distinct LRRK2 PD risk variants (Fig. S3A,B). As assessed by immunoblotting, transient expression of the pathogenic Y1699C-LRRK2 mutant caused a detectable increase in Rab10 phosphorylation as compared to wildtype LRRK2, whilst no significant differences were observed when expressing the various LRRK2 risk variants (Fig. S3C). However, increased accumulation of phospho-Rab10 in individually transfected cells could be detected by immunocytochemistry when expressing pathogenic LRRK2 or the various risk variants, and such accumulation was reverted by MLi2 in all cases (Fig. S3D).

Conversely, to analyze the effect of the R1398H mutation in LRRK2 which is protective against PD (Tan et al., 2010; Chen et al., 2011; Ross et al., 2011), we introduced it into either wildtype or pathogenic LRRK2 constructs. Transient expression of pathogenic G2019S, R1441C, Y1699C, N1437H, or I2020T LRRK2 mutants caused a significant deficit in centrosomal cohesion that was attenuated by introduction of the protective R1398H variant in all cases (Fig. S4). Similar results were obtained when introducing synthetic mutations (R1398L or R1398L/T1343V) described to alter Rab10 phosphorylation by modulating LRRK2 GTP binding and hydrolysis (Xiong et al., 2010; Biosa et al., 2013; Blanca Ramírez et al., 2017; Nguyen et al., 2020) (Fig. S5). Thus, risk or protective LRRK2 variants can either negatively or positively impact upon the centrosomal cohesion deficits mediated by the LRRK2 kinase activity, at least under overexpression conditions in HEK293T cells as employed here.

### Centrosomal cohesion deficits mediated by pathogenic LRRK2 are independent of Rab12, Rab35, Rab43 and RILPL2 in A549 cells

Our previous studies have implicated Rab8 and Rab10 in the centrosomal deficits mediated by pathogenic LRRK2 (Dhekne et al., 2018; Lara Ordóñez et al., 2019). To study the potential role of the LRRK2 kinase substrates Rab12, Rab35 or Rab43, we employed A549 cells where these proteins were knocked out using CRISPR-Cas9 (Steger et al., 2017) (Fig. S6A,B). Expression of pathogenic LRRK2 in wildtype A549 cells caused a pronounced deficit in centrosomal cohesion (Fig. S6C), and similar deficits were observed when pathogenic LRRK2 was expressed in A549 cells deficient in either Rab12, Rab35 or Rab43 (Fig. S6B-G). In all cases, the deficits were reverted by transient application of MLi2 and were not detected when expressing wildtype LRRK2 or a kinase-inactive LRRK2 mutant. Furthermore, and as assessed by immunoblot analysis, expression of pathogenic LRRK2 caused similar increases in the levels of phospho-Rab10 in wildtype cells and in cells deficient in either Rab12, Rab35, or Rab43 (Fig. S6H). Finally, centrosomal cohesion deficits mediated by pathogenic LRRK2 were also observed in A549 RILPL2 knockout cells (Fig. S7). These data indicate that the centrosomal cohesion deficits mediated by pathogenic LRRK2 are not dependent on the presence of Rab12, Rab35, Rab43 or RILPL2, at least in the cell system analyzed here.

### Perinuclear localization of phospho-Rab10 is crucial for the centrosomal cohesion deficits mediated by pathogenic LRRK2

Wildtype A549 cells transfected with pathogenic LRRK2 displayed prominent phospho-Rab10 staining in a perinuclear area and in tubular structures, and a similar staining was observed in RILPL2 knockout cells (Fig. S8). In contrast, and as previously reported (Dhekne et al., 2018), RILPL1 knockout cells transfected with pathogenic LRRK2 showed a diminished perinuclear distribution of phospho-Rab10 accompanied by punctate staining throughout the cytosol (Fig. S8).

**Fig. 3.**
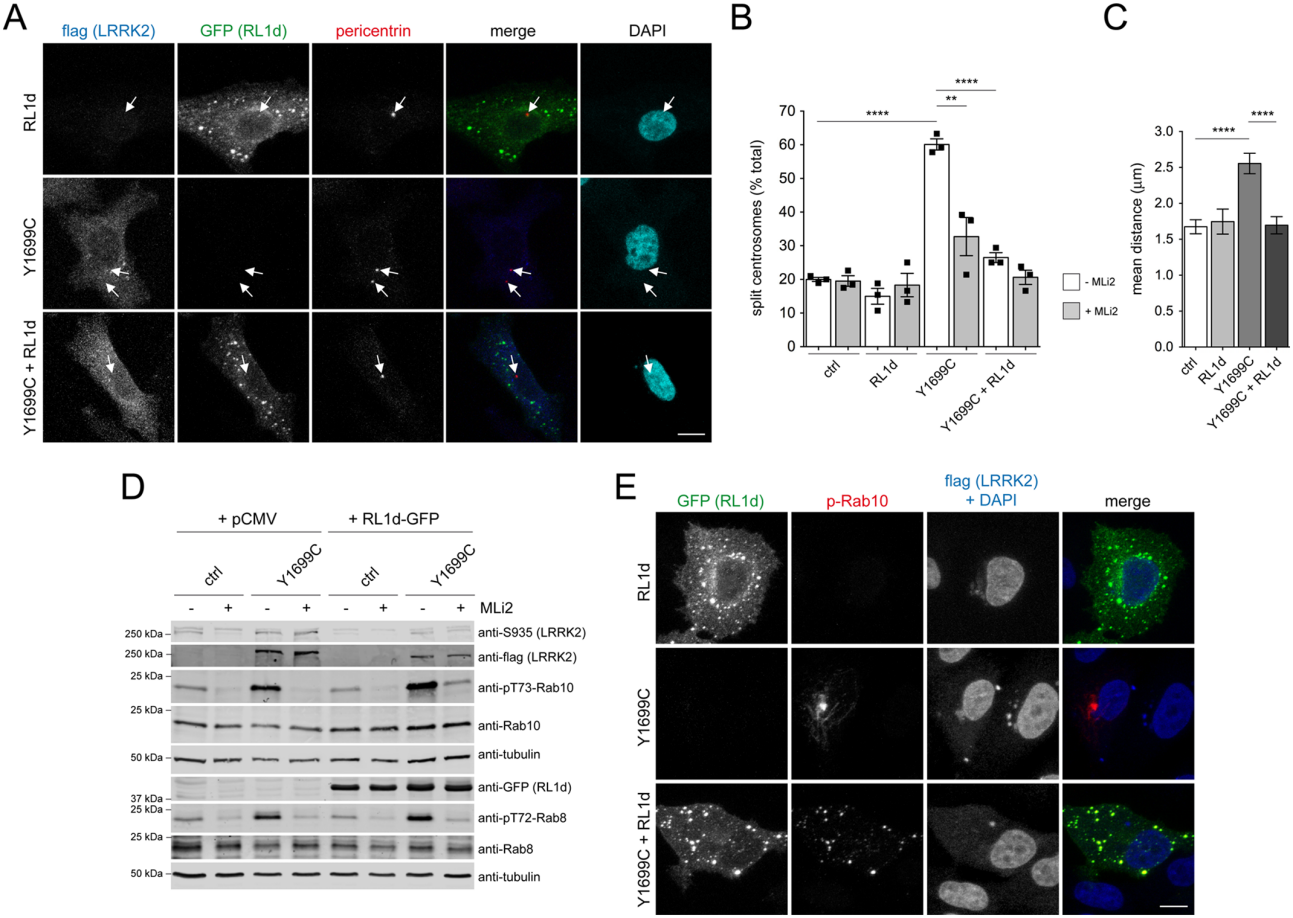
**Expression of C-terminal RILPL1 reverts the LRRK2-mediated centrosomal cohesion deficits and redistributes phospho-Rab10.** (A) Example of A549 cells transfected with the C-terminal region of RILPL1 (RL1d-GFP), flag-tagged Y1699C mutant LRRK2 or both, and stained with antibodies against flag (Alexa-594 secondary antibody; pseudocolored in blue), pericentrin (Alexa-647 secondary antibody; red) and with DAPI. Arrows point to centrosomes in transfected cells. Scale bar: 10 µm. (B) Quantification of the percentage of cells displaying duplicated split centrosomes transfected with either pCMV (ctrl), RL1d-GFP, flag-tagged Y1699C LRRK2, or with both, and either left untreated or incubated with MLi2 (200 nM, 2 h) prior to immunocytochemistry. Bars represent mean±s.e.m. (*n*=3 experiments); ****P<0.001; **P<0.01. (C) Cells transfected with the indicated constructs were processed for immunocytochemistry, and the distances between duplicated centrosomes were quantified from around 50 cells each. ****P<0.001. (D) Cells were co-transfected with the indicated constructs, left untreated or incubated with MLi2 (200 nM, 2 h) as indicated, and extracts were blotted for flag-tagged LRRK2, phosphorylated LRRK2 (S935), pT73-Rab10, total Rab10, pT72-Rab8a, total Rab8a or tubulin as loading control. (E) Cells were transfected with the indicated constructs and stained with antibodies against phospho-Rab10 (Alexa-594 secondary antibody, red), flag (Alexa-405 secondary antibody, blue) and DAPI. Scale bar: 10 µm.

To study the importance of the subcellular localization of phospho-Rab10 for the LRRK2-mediated centrosomal cohesion deficits, we expressed the C-terminal half of RILPL1 (RL1d-GFP) reported to be responsible for its interaction with the phosphorylated Rab proteins (Steger et al., 2017; Dhekne et al., 2018). When expressed in A549 cells, RL1d-GFP displayed a punctate as well as cytosolic localization (Fig. 3A). Strikingly, RL1d-GFP expression completely reverted the centrosomal cohesion phenotype induced by pathogenic LRRK2, while not displaying an effect when expressed on its own (Fig. 3A-C). Co-expression of RL1d-GFP with pathogenic LRRK2 did not decrease the total levels of phospho-Rab8 or phospho-Rab10 as assessed by immunoblot analysis (Fig. 3D). Rather, whilst pathogenic LRRK2 caused a pronounced perinuclear accumulation of phospho-Rab10, co-expression with RL1d-GFP caused the redistribution of phospho-Rab10 to cytosolic RL1d-GFP-positive punctae (Fig. 3E), which did not colocalize with early endosomal or lysosomal markers (Fig. S9). These data indicate that it is the perinuclear accumulation of phospho-Rab10, rather than phospho-Rab10 levels *per se*, that is required to cause the cohesion deficits mediated by pathogenic LRRK2.

### RILPL1 localizes to subdistal appendages of the mother centriole

**Fig. 4.**
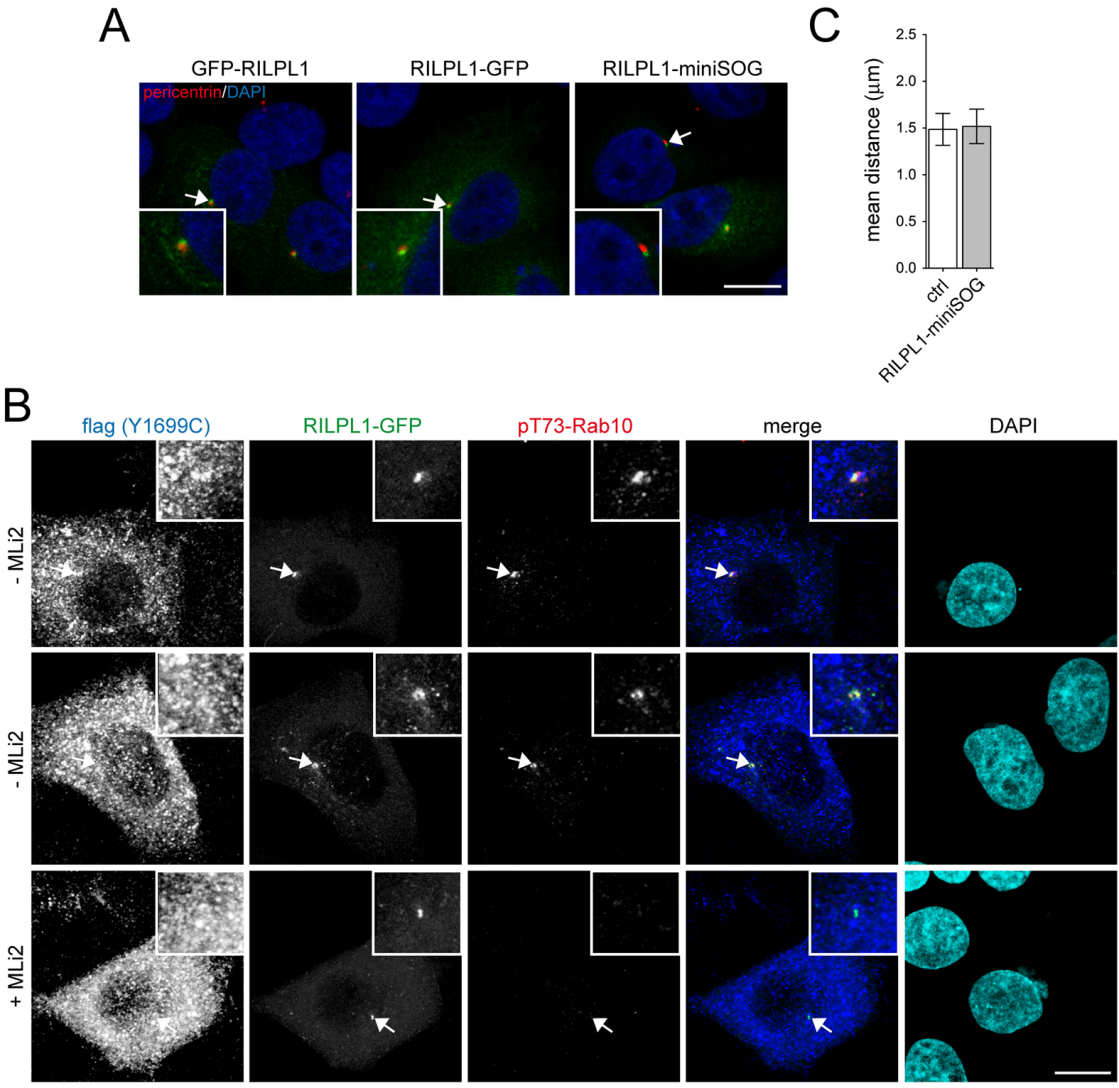
**RILPL1 localizes to the centrosome and recruits phospho-Rab10 to this location.** (A) Example of A549 cells transfected with GFP-RILPL1, RILPL1-GFP or RILPL1-miniSOG, and stained with antibody against pericentrin (red) and with DAPI. Scale bar: 10 µm. (B) Examples of A549 cells co-transfected with flag-tagged Y1699C-LRRK2 and RILPL1-GFP, left untreated or incubated with MLi2 (200 nM, 2 h) as indicated, and stained with an antibody against flag (Alexa-647 secondary antibody; pseudocolored in blue), an antibody against phospho-Rab10 (Alexa-555 secondary antibody; red) and with DAPI (cyan). Arrows point to RILPL1-GFP localization. Scale bar: 10 µm. (C) A549 cells were transfected with RILPL1-miniSOG and processed for immunocytochemistry, and the distances between duplicated centrosomes quantified from around 30 non-transfected and transfected cells. Bars represent mean±s.e.m.

In the absence of synthetically induced lysosomal damage, pathogenic LRRK2 causes accumulation of phosphorylated Rab10 in a pericentrosomal location (Dhekne et al., 2018; Lara Ordóñez et al., 2019). Conversely, RILPL1 has been reported to associate with the centrosome (Dhekne et al., 2018). We corroborated the centrosomal localization of endogenous RILPL1, and the colocalization of RILPL1 with endogenous phospho-Rabs in HEK293 T cells transfected with pathogenic LRRK2 by employing two distinct anti-RILPL1 antibodies (Fig. S10). Since N-terminally or C-terminally GFP-tagged RILPL1 also displayed a centrosomal localization when expressed in A549 cells (Fig. 4A), we co-expressed tagged RILPL1 along with flag-tagged Y1699C-LRRK2. Under those conditions, the phospho-Rab10 signal also extensively colocalized with RILPL1, consistent with the notion that RILPL1 recruits phospho-Rab10 to the centrosome (Fig. 4B).

**Fig. 5.**
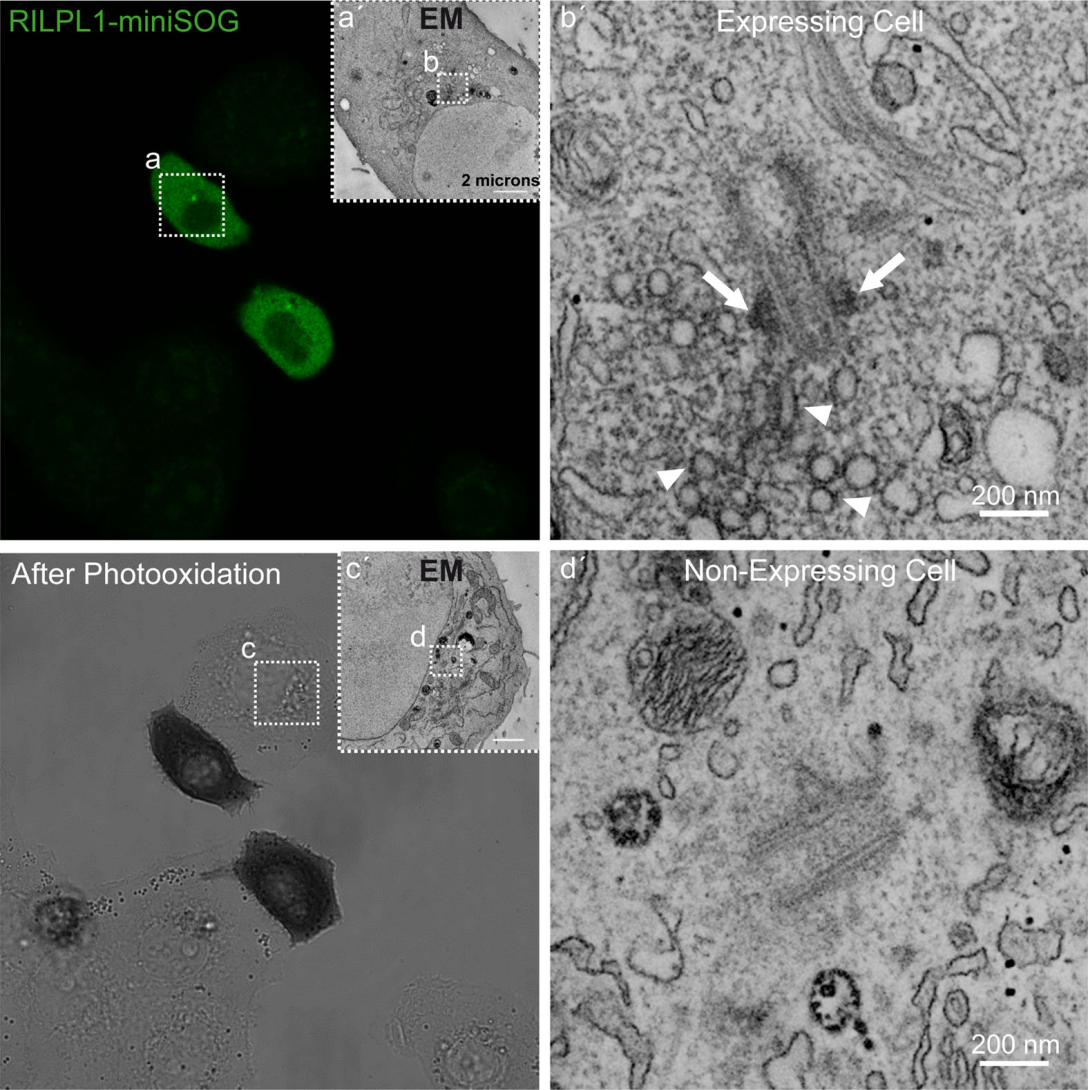
**RILPL1 localizes to the subdistal appendage of the mother centriole.** Correlated light and EM imaging of RILPL1-miniSOG. Transfected A549 cells were revealed first by confocal fluorescence and then by transmitted light imaging following DAB photooxidation, where an optically dense reaction product was observed in the two expressing cells. To better discriminate the DAB precipitate, the tannic acid and uranyl acetate stainings were omitted. Low magnification TEM image of the RILPL1-miniSOG-expressing cell (A′) corresponds to the same area indicated by the white square (A); similarly, the high magnification image (B′) corresponds to the same area indicated by the white square (B). White arrows point at DAB-labeled subdistal appendages of the mother centriole; arrowheads point at DAB-labeled pericentrosomal vesicles. For comparison, the low magnification TEM image of the non-expressing cell (C′) corresponds to the same area indicated by the white square (C); the high magnification image (D′) corresponds to the same area indicated by the white square (D). No labeling was observed at the subdistal appendages in the control, non-expressing cell. These observations were confirmed in cells (RILPL1-miniSOG-expressing and control non-expressing) from three different areas.

We next fused RILPL1 to miniSOG (RILPL1-miniSOG), a tag suitable for correlative light and electron microscopy (CLEM) (Shu et al., 2011; Boassa et al., 2013), and we assured that transient expression of RILPL1-miniSOG did not interfere with proper centrosome cohesion (Fig. 4C). In order to image the pericentrosomal localization of RILPL1 with high spatial resolution, we performed miniSOG-induced DAB oxidation which generated a localized polymeric precipitate that could be readily identified by CLEM (Fig. 5). Using STEM tomography (for which we combined a multiple-tilt tomography approach with the scanning mode of a TEM), we determined that RILPL1 was localized to the subdistal appendage of the mother centriole, hinting that the interaction between phospho-Rab10 and RILPL1 occurs at this location. The specific DAB labelling was clearly distinguishable in RILPL1-miniSOG expressing A549 cells as compared to adjacent non-expressing cells that were exposed to the same processing within the photooxidized area (Fig. 5). An accumulation of DAB-labeled pericentrosomal vesicles was also observed, but only in cells expressing RILPL1, indicating that such vesicle accumulation may have been induced by RILPL1 overexpression. These data suggest that RILPL1 localizes to the subdistal appendage of the mother centriole, and that the phospho-Rab10/RILPL1 interaction may occur at this location.

### Ciliogenesis and centrosomal cohesion deficits in MEFs expressing endogenous levels of R1441C-LRRK2 or vps35-D620N

**Fig. 6.**
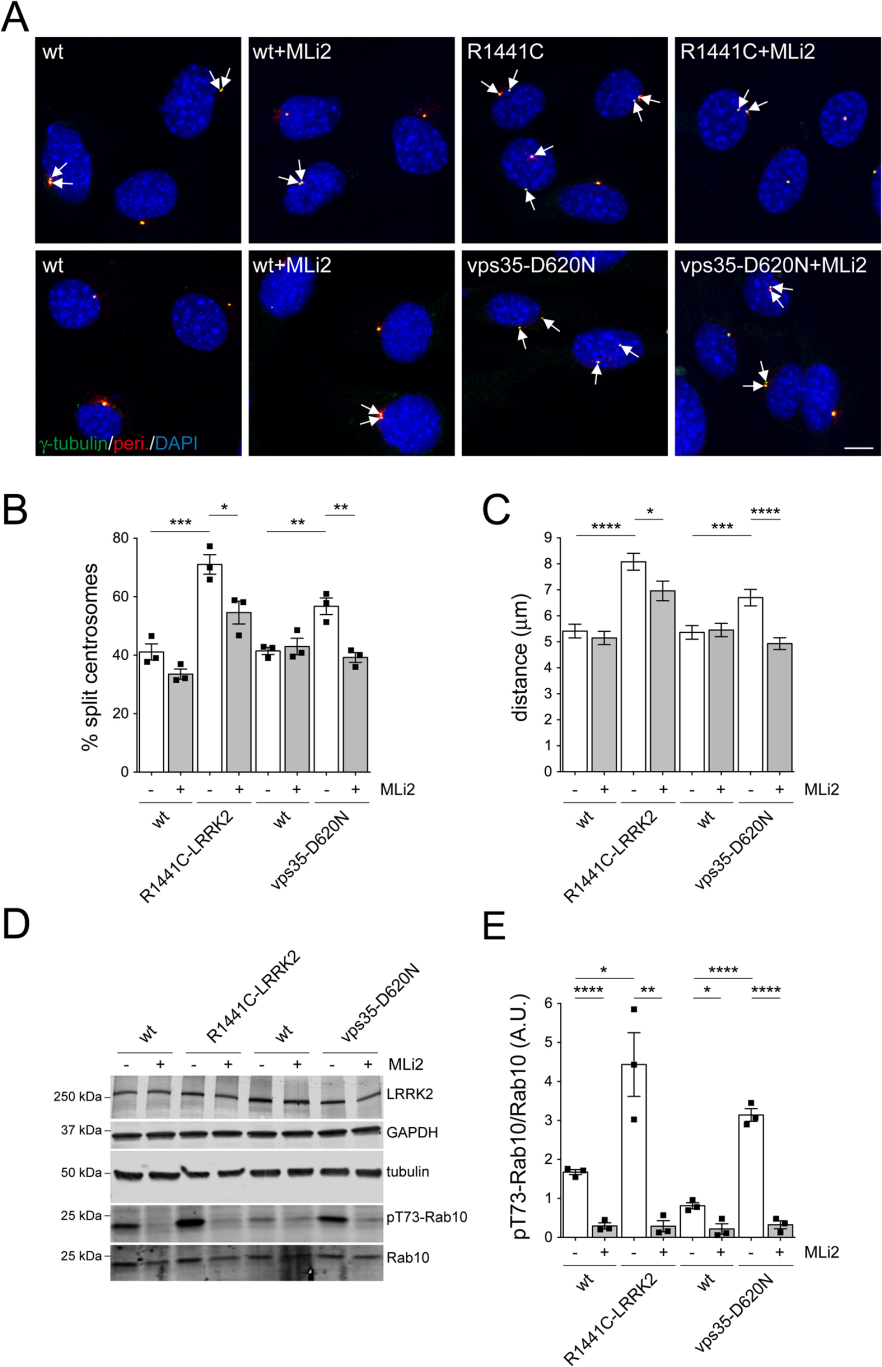
**Kinase activity-mediated centrosomal cohesion deficits in R1441C-LRRK2 and vps35-D620N MEF cells.** (A) Representative images of wildtype (wt) littermate and R1441C-LRRK2 MEFs, and respective wt littermate and vps35-D620N MEFs treated with either DMSO or with MLi2 (200 nM, 2 h) before processing for immunocytochemistry with antibodies against two centrosomal markers (pericentrin and γ-tubulin) and with DAPI. Arrows point to duplicated centrosomes. Scale bar: 10 µm. (B) Quantification of the percentage of cells with duplicated split centrosomes in wt and R1441C-LRRK2 MEFs, and wt and vps35-D620N MEFs in the absence or presence of MLi2. Bars represent mean±s.e.m. (*n*=3 independent experiments); ****P<0.001; ***P<0.005; **P<0.01; *P<0.05. (C) Quantification of the distances between duplicated centrosomes in the absence or presence of MLi2. Bars represent mean±s.e.m. (*n*=60-130 cells with duplicated centrosomes per genotype); ****P<0.001; ***P<0.005; *P<0.05. (D) MEFs as indicated were treated with DMSO or MLi2 (200 nM, 2 h), and extracts blotted for LRRK2 and GAPDH, or for phospho-Rab10 (pT73-Rab10), Rab10 and tubulin as the loading control. (E) Quantification of pT73-Rab10/Rab10 levels from Western blots of the type depicted in (D). Bars depict mean±s.e.m. (*n*=3 independent extracts per genotype); ****P<0.001; **P<0.01; *P<0.05.

**Fig. 7.**
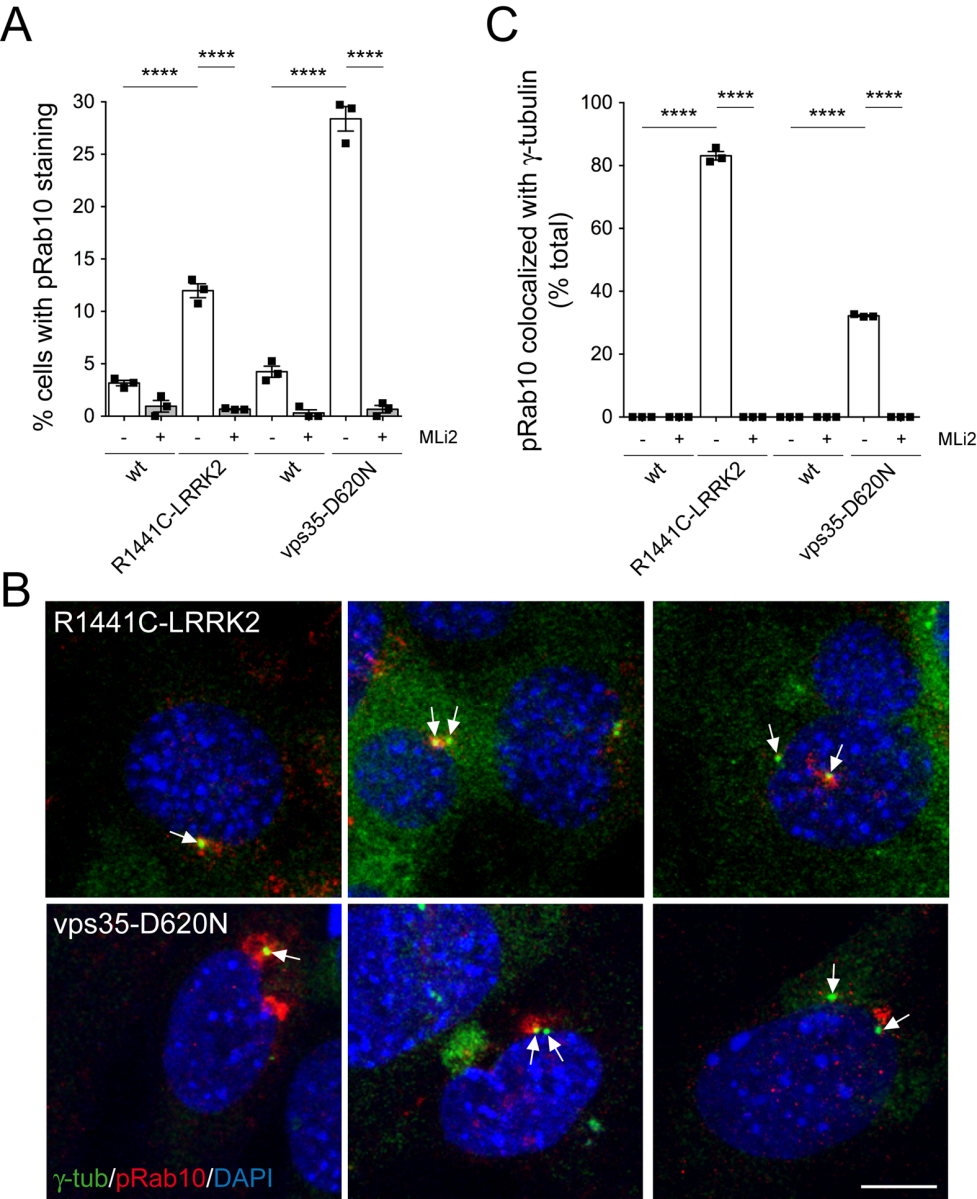
**LRRK2 kinase-mediated centrosomal phospho-Rab10 accumulation in R1441C-LRRK2 and vps35-D620N MEF cells.** (A) Quantification of the percentage of wildtype (wt) littermate and R1441C-LRRK2 MEFs, and respective wt littermate and vps35-D620N MEFs displaying phospho-Rab10 staining in either the absence or presence of MLi2 (200 nM, 2 h). Between 70 and 160 random cells were scored per condition and experiment. Bars represent mean±s.e.m. (*n*=3 independent experiments); ****P<0.001. (B) Cells of the indicated genotype were stained with antibodies against a centrosomal marker (γ-tubulin), phospho-Rab10 and DAPI. Arrows point to centrosomes. Note that as observed for endogenous RILPL1 (see Fig. S9), phospho-Rab10 only associates with one of the two duplicated centrosomes, consistent with its recruitment to the subdistal appendage of the ‘older’ mother centriole. Scale bar: 10 µm. (C) Quantification of the percentage of cells of the indicated genotype displaying colocalization of phospho-Rab10 with γ-tubulin. Bars represent mean±s.e.m. (*n*=3 independent experiments); ****P<0.001.

Previous studies reported ciliogenesis deficits in mouse embryonic fibroblasts (MEFs) derived from R1441C-LRRK2 mice (Sobu et al., 2021), and we observed similar ciliogenesis deficits in MEFs from heterozygous vps35-D620N knockin mice as compared to their respective wildtype littermate controls (Fig. S11). Importantly, R1441C-LRRK2 and vps35-D620N MEFs also both displayed centrosomal cohesion deficits as evidenced by an increase in the mean distance between duplicated centrosomes (Fig. 6A-C). These cohesion deficits were significantly reduced (for R1441C-LRRK2) or fully reverted (for vps35-D620N) by MLi2 (Fig. 6B,C) and correlated with a LRRK2 kinase activity-mediated increase in the levels of phospho-Rab10 as assessed by immunoblotting (Fig. 6D,E). The R1441C-LRRK2 and vps35-D620N MEFs showed an increase in phospho-Rab10 staining as compared to their wildtype littermate controls which was abolished by MLi2 treatment (Fig. 7A). Phospho-Rab10 staining was heterogenous, with only a subset of R1441C-LRRK2 or vps35-D620N MEFs displaying a detectable signal (Fig. 7A). However, in the vast majority of cells positive for phospho-Rab10, this signal colocalized with a centrosomal marker (Fig. 7B,C). Colocalization was observed in cells with one centrosome (G1) as well as in cells with duplicated centrosomes (G2), irrespective of whether these were properly held together or displayed a cohesion deficit (Fig. 7B). Furthermore, in cells with duplicated centrosomes, phospho-Rab10 colocalized with only one of the two centrosomes, consistent with its RILPL1-mediated localization to the subdistal appendage of the (older) mother centriole. Thus, increased LRRK2 kinase activity either due to pathogenic LRRK2 mutations or vps35-D620N-mediated LRRK2 activation causes centrosomal cohesion deficits and centriolar phospho-Rab10 accumulation in endogenous cellular contexts.

### Pathogenic LRRK2 and vps35-D620N cause deficits in cell polarization

**Fig. 8.**
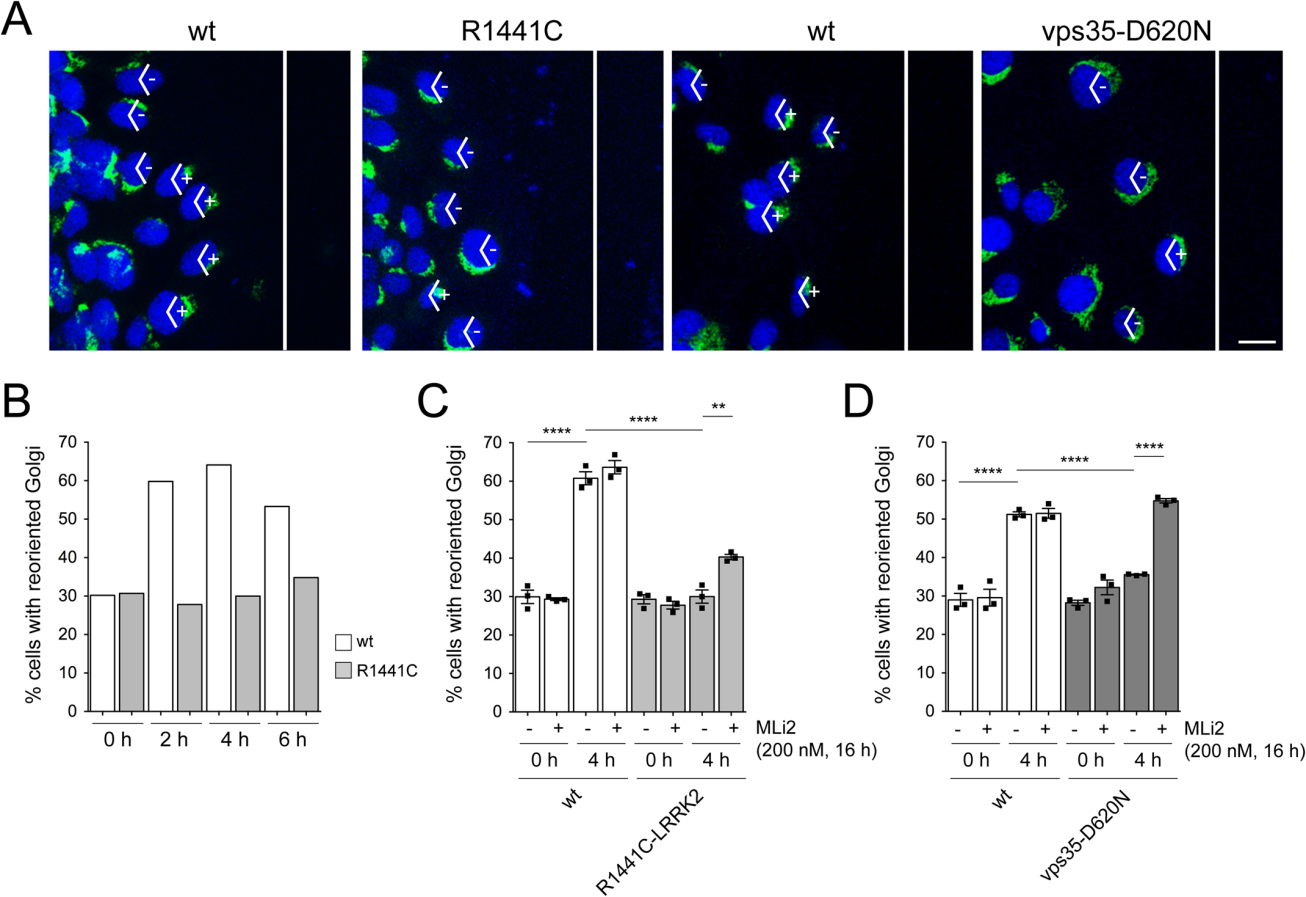
**Kinase activity-mediated deficits in cell polarization in R1441C-LRRK2 and vps35-D620N MEF cells.** (A) Example of reorientation of the Golgi in wt littermate and R1441C-LRRK2 MEFs, or wt littermate and vps35-D620N MEFs 4 h after wounding. The white lines indicate scratch orientation, and cells were stained with an antibody against a Golgi protein (ACBD3) and DAPI. Angles (120°) are labeled as having oriented (+) or not oriented (−) Golgi complex for the first row of cells facing the scratch wound. Scale bar: 15 µm. (B) Quantification of Golgi reorientation in wt littermate and R1441C-LRRK2 MEFs immediately after (*t*=0 h) or at various time points after generating the wound. Random orientation is expected to be 33%. *N*>100 cells in the first row of cells facing the scratch were quantified for each timepoint and genotype. (C) Quantification of Golgi reorientation in wt littermate and R1441C-LRRK2 MEFs in either the absence or presence of MLi2 (200 nM, 16 h) and either at *t*=0 h or *t*=4 h after generating the wound. Bars represent mean±s.e.m. (*n*=3 independent experiments); ****P<0.001; **P<0.01. (D) Quantification of Golgi reorientation in wt littermate and vps35-D620N MEFs in either the absence or presence of MLi2 (200 nM, 16 h) and either at *t*=0 h or *t*=4 h after generating the wound. Bars represent mean±s.e.m. (*n*=3 independent experiments); ****P<0.001.

Depletion of proteins critical for centrosome cohesion does not lead to cell cycle arrest (Fry et al., 1998; Mayor et al., 2000; Faragher and Fry, 2003; Flanagan et al., 2017), but can cause profound deficits cell polarization (Floriot et al., 2015; Panic et al., 2015), a prerequisite for directional cell migration whereby the centrosome and Golgi complex reorient towards the leading edge of a migrating cell (Etienne-Manneville and Hall, 2001; Hurtado et al., 2011; Elric and Etienne-Manneville, 2014; Etienne-Manneville, 2014). Therefore, we wondered whether mutant LRRK2 or mutant vps35 may display deficits in cell polarization associated with altered centrosome and Golgi positioning and mediated by the LRRK2 kinase activity. We first confirmed the close juxtaposition between the centrosome and Golgi complex in wildtype as well as in R1441C-LRRK2 and vps35-D620N MEFs, respectively (Fig. S12A). We next performed scratch wound assays with the different MEF lines and scored cells as polarized when the Golgi was located within a 120° angle facing the wound (Etienne-Manneville and Hall, 2001) (Fig. 8A). Around 60% of wildtype cells had already reoriented the Golgi at 2 h after wounding, with no further increase obtained at later timepoints (Fig. 8B). After 4 h of wounding, around 60% of control cells had reoriented the Golgi, whilst the R1441C-LRRK2 or vps35-D620N MEFs were entirely deficient in cell polarization (Fig. 8C,D). MLi2 treatment fully reversed the cell polarization deficit in the vps35-D620N MEFs, while only a partial reversal was observed in the R1441C-LRRK2 MEFs (Fig. 8C,D). Analysis of Golgi morphology in the distinct MEF lines revealed a small percentage of R1441C-LRRK2 cells displaying a fragmented Golgi, and a larger percentage of cells displaying two spatially distinct Golgi stacks, a phenotype that was not reverted by MLi2 and was not observed in the vps35-D620N MEFs (Fig. S12B,C). The presence of two spatially distinct Golgi stacks has been previously reported in individual cells with very pronounced deficits in centrosomal cohesion (Panic et al., 2015), and may explain why MLi2 only partially reverted the cohesion and cell polarization deficits in the R1441C-LRRK2 MEFs. Together, these data indicate that both pathogenic LRRK2 and vps35-D620N cause LRRK2 kinase-mediated centrosomal deficits which impair appropriate cell polarization, a prerequisite for cells to properly respond to directional migration signals.

## DISCUSSION

Pathogenic LRRK2 causes deficits in the proper cohesion of duplicated centrosomes which is dependent on RILPL1 and on the LRRK2-phosphorylated Rab8 and Rab10 proteins (Madero-Pérez et al., 2018; Fernández et al., 2019; Lara Ordóñez et al., 2019). In this study, we show that centrosomal cohesion deficits are commonly observed when altering the LRRK2 kinase signaling pathway by other means. A point mutation in vps35 which causes autosomal-dominant PD and activates LRRK2 (Vilariño-Güell et al., 2011; Zimprich et al., 2011; Sharma et al., 2012; Mir et al., 2018) also causes a pronounced cohesion deficit, including in cells expressing endogenous levels of mutant vps35. Knockout of PPM1H, the phosphatase specific for the dephosphorylation of Rab8 and Rab10 (Berndsen et al., 2019), impairs centrosomal cohesion in the presence of LRRK2 expression. These findings exclude a potential role for other LRRK2 kinase substrates and suggest that phospho-Rab8/10 are both necessary and sufficient to cause the centrosomal phenotypes. Finally, cohesion alterations are also observed when expressing LRRK2 risk variants. In all cases, the deficits are reverted by MLi2, indicating that they are LRRK2-kinase-activity-mediated. These data provide strong evidence for the importance of the LRRK2 kinase signaling pathway in regulating centrosomal cohesion in dividing cells also under endogenous expression conditions. The centrosomal defects require accumulation of phospho-Rab8/10 in a manner dependent on RILPL1 but are independent of the presence of other LRRK2 kinase substrates (Rab12, Rab35, Rab43), or RILPL2. Thus, RILPL1 functions as a key player for the LRRK2-mediated centrosomal deficits by enabling the recruitment of phospho-Rab8/10 to the subdistal appendage of the mother centriole. Short-term application of a LRRK2 kinase inhibitor reverts the increase in phospho-Rab8/10, and this in turn leads to a rapid reversal of the centrosomal cohesion deficits. Such dynamic behavior is consistent with the direct RILPL1-mediated recruitment of phospho-Rab8/10 to a centriolar location to cause an increase in the distance between duplicated centrosomes. It further predicts that the underlying mechanism may involve the dynamic displacement of protein(s) necessary to keep the duplicated centrosomes in close proximity to each other (Fdez et al., 2022), perhaps via steric hindrance.

Deep proteomics analysis of mammalian cell lines shows similar expression levels for a given Rab protein in HEK293T versus A549 cells (Geiger et al., 2012; Schaab et al., 2012) and copy number proteomics reveals comparable expression levels also in murine embryonic fibroblasts (MEFs) (Nirujogi et al., 2021). Therefore, the three cell types employed here express similar levels of the distinct LRRK2 kinase substrates (Rab8a, Rab10, Rab12, Rab29, Rab35, Rab43), with Rab8a and Rab10 in at least 10-fold excess over the others. Such quantitative proteomics further indicates that HEK293T, A549 and MEF cells contain 10-20-fold more Rab8a and Rab10 than RILPL1 (Nirujogi et al., 2021). Therefore, the LRRK2 kinase activity is able to profoundly affect the extent to which RILPL1 is complexed with phospho-Rab8/10 to bring about the centrosomal deficits, at least in the cell types studied here.

As determined by CLEM, RILPL1 localizes to the subdistal appendage of the mother centriole in wildtype A549 cells. Phospho-Rab10 staining largely overlaps with tagged RILPL1 in A549 cells over-expressing pathogenic LRRK2 and partially overlaps with a centrosomal marker in MEFs endogenously expressing pathogenic LRRK2. Since it is the membrane-bound active form of Rab10 which is phosphorylated by LRRK2 (Liu et al., 2018; Gomez et al., 2019; Lara Ordóñez et al., 2019; Fdez et al., 2022), it will be interesting to determine whether membrane-bound vesicular structures containing phospho-Rab10 accumulate at the subdistal appendage of the mother centriole in pathogenic LRRK2-expressing cells. In addition, and given recent reports of the localization of active Rab10 at the mother centriole in a manner regulated by the GDP/GTP exchange factor DENND2B (Kumar et al., 2022), future studies should address whether the pathogenic LRRK2-mediated centriolar phospho-Rab10 accumulation and the resulting cohesion deficits are modulated by DENND2B.

We have previously reported centrosomal cohesion deficits in lymphoblastoid cell lines from G2019S LRRK2-PD patients as compared to healthy controls, and such deficits were also observed in a subset of sporadic PD patients (Madero-Pérez et al., 2018; Fernández et al., 2019). In future experiments, it will be interesting to determine whether centrosomal deficits can be detected in peripheral cells from PD patients harboring LRRK2 risk variants or mutations in vps35. In addition, our study shows that expression of the N2081D LRRK2 mutant causes kinase activity-mediated centrosomal cohesion deficits. Since the N2081D LRRK2 mutation confers risk for PD as well as for Crohn's disease (Hui et al., 2018), further studies are warranted to probe for centrosomal deficits in peripheral cells from Crohn's disease patients, as this may aid in stratifying patients benefitting from LRRK2 kinase inhibitor therapeutics in clinical studies.

Recent work has shown that inducing lysosomal damage causes recruitment of wildtype LRRK2 to lysosomes, followed by the lysosomal accumulation of phospho-Rab10 (Eguchi et al., 2018; Bonet-Ponce et al., 2020; Herbst et al., 2020; Kuwahara et al., 2020). Conversely, mitochondrial depolarization causes the mitochondrial accumulation of Rab10 to facilitate mitophagy, and such accumulation is impaired in the context of pathogenic LRRK2 (Wauters et al., 2020). Importantly, our studies were performed in the absence of treatments to induce lysosomal damage or mitochondrial depolarization. Under such normal physiological conditions, we find that phospho-Rab10 localizes at the centrosome to cause the centrosomal cohesion deficits. In future experiments, it will be interesting to determine how triggers that lead to lysosomal or mitochondrial damage might impact upon the centrosomal deficits as described here. In either case, our data indicate that it is the RILPL1-mediated localization of phospho-Rab10 which is responsible for the cohesion deficits due to pathogenic LRRK2. Interestingly, the expression of a C-terminal fragment of RILPL1 that localizes to cytosolic punctate structures reverts the cohesion deficits by redistributing phospho-Rab10 from its centrosomal location to those structures, but does not alter the total levels of phospho-Rab10 as assessed by Western blot analysis. Therefore, the subcellular location of phospho-Rab10, rather than total phospho-Rab10 levels, is relevant for our understanding of pathogenic LRRK2 action in a given cellular context.

Previous studies have shown that interfering with centrosomal cohesion does not affect cell doubling time (Mayor et al., 2000; Flanagan et al., 2017) but can cause pronounced deficits in directional cell migration (Floriot et al., 2015; Panic et al., 2015). Here, we find that the centrosomal cohesion deficits due to pathogenic LRRK2 or vps35 in MEF cells are associated with kinase activity-mediated defects in cell polarization which is required for directional cell migration. Pathogenic LRRK2 has been reported to reduce microglial motility, which may impair microglial relocalization due to local insults and in this manner contribute to LRRK2-associated neurodegeneration (Choi et al., 2015). In future, it will be interesting to determine whether the LRRK2 kinase-mediated deficits in microglial motility are also due to a centriolar phospho-Rab10/RILPL1 complex.

Altogether, our data demonstrate that pathogenic LRRK2 mutations, LRRK2 risk variants and modulators of the LRRK2 signaling pathway all converge upon causing centrosomal cohesion defects. These deficits are dependent on RILPL1 and directly mediated by the centrosomal accumulation of phospho-Rab10. The localization of RILPL1 implicates the subdistal appendage of the mother centriole as the prime site of action for the LRRK2-mediated phospho-Rab10 accumulation, with downstream effects on centrosomal cohesion and cell polarization as described here.

## MATERIALS AND METHODS

### DNA constructs and site-directed mutagenesis

GFP-tagged human wildtype LRRK2, pathogenic mutant LRRK2 (G2019S, R1441C, R1441G, Y1699C, N1437H, I2020T) kinase-inactive mutant LRRK2 (K1906M), as well as all mutant LRRK2 constructs containing R1398H, R1398L, T1343V, or R1398L/T1343V have previously been described (Blanca Ramírez et al., 2017; Madero-Pérez et al., 2018; Lara Ordóñez et al., 2019). The T1410A, R1628P, S1647T, N2081D and G2385R mutant GFP-tagged LRRK2 constructs were generated by site-directed mutagenesis (QuikChange, Stratagene), and identity of all constructs verified by sequencing of the entire coding region. Flag-tagged human wildtype and mutant LRRK2 constructs, as well as N-terminally or C-terminally GFP-tagged human RILPL1 constructs have been previously described (Lara Ordóñez et al., 2019). RILPL1-miniSOG-HA construct was generated using Gibson Assembly Master Mix (New England Biolabs), and identity of construct verified by sequencing of the entire coding region. N-terminally HA-tagged human vps35, vps35-D620N, vps35-L774M, vps35-M57I, and C-terminal half of human RILPL1 [E280-end] tagged at the C-terminus with eGFP (RL1d-GFP) (Steger et al., 2017) were generous gifts from Dario Alessi (University of Dundee, UK), and are available at https://mrcppureagents.dundee.ac.uk/. For transient transfections of mammalian cells, all DNA constructs were prepared from bacterial cultures grown at 37°C using PureYield^TM^ Plasmid Midiprep System (Promega) according to manufacturer's instructions.

### Cell culture and transfections

HEK293T cells (ATCC CRL-3216) were cultured in full medium (Dulbecco's modified Eagle's medium, DMEM, containing low glucose and 10% fetal bovine serum, non-essential amino acids, 100 U/ml penicillin and 100 µg/ml streptomycin) and transfected at 80% confluence with 1 µg of LRRK2 constructs (and 100 ng of HA-tagged vps35 constructs where indicated) and 3 µl of LipoD293^TM^ Transfection Reagent (SignaGen Laboratories) per well of a 12-well plate overnight. The next day, cells were split to 25% confluence onto poly-L-lysine-coated coverslips and subjected to immunocytochemistry or Western blot analysis 48 h after transfection.

The various wildtype and CRISPR-Cas9 knockout A549 cells (Rab10-KO, Rab12-KO, Rab35-KO, Rab43-KO, RILPL1-KO, RILPL2-KO, vps35-KO and PPM1H-KO) were generous gifts from Dario Alessi and have been previously described (Ito et al., 2016; Steger et al., 2017; Dhekne et al., 2018; Mir et al., 2018; Purlyte et al., 2018; Berndsen et al., 2019; Dhekne et al., 2021). Cells were cultured in DMEM containing high glucose without glutamine, and supplemented with 10% fetal bovine serum, 2 mM L-glutamine, 100 U/ml of penicillin and 100 µg/ml of streptomycin. Cells were subcultured at a ratio of 1:6-1:10 twice a week and transfected at 90% confluence. Cells were either transfected with 1 µg of LRRK2 constructs, co-transfected with 1 µg of LRRK2 and 100 ng RL1d-GFP construct, 1 µg of LRRK2 and 100 ng of HA-tagged vps35 constructs, or co-transfected with 1 µg of pCMV and 100 ng of GFP-RILPL1, RILPL1-GFP or RILPL1-miniSOG constructs, along with 4 µl of LipoD293^TM^ Transfection Reagent (SignaGen Laboratories) per well of a 12-well plate. Transfection media was replaced with full media after 5 h, cells were split 1:4 the next day and processed for immunocytochemistry or Western blotting 48 h after transfection.

Littermate matched wildtype and homozygous LRRK2-R1441C knockin MEFs generated from mice at E12.5 (resulting from crosses between heterozygous LRRK2-R1441C/wildtype mice maintained on a C57BL/6J background) were spontaneously immortalized by prolonged passaging, and were a generous gift from Dario Alessi (Steger et al., 2017). Similarly, wildtype and heterozygous vps35-D620N knockin MEFs were isolated from littermate-matched mouse embryos at E12.5 (resulting from crosses between heterozygous vps35-D620N/wildtype mice) were spontaneously immortalized by prolonged passaging, and were a generous gift from Dario Alessi (Mir et al., 2018). In all cases, cells were grown in full medium consisting of DMEM containing high glucose (Gibco, 11960-044), 10% fetal bovine serum (Gibco, 10438-026), 1 mM sodium pyruvate (Gibco, 11360-070), non-essential amino acids (Gibco, 11140-050), 2 mM L-glutamine (Gibco, 25030-081), 100 U/ml penicillin and 100 µg/ml streptomycin (Gibco, 15140-122). Cells were passaged at around 90% confluence to a ratio of 1:10 for general maintenance, with media exchanged every 2 days.

In all cases, cells were grown at 37°C and 5% CO_2_ in a humidified atmosphere, and all lines were regularly tested for mycoplasma contamination. Co-transfection efficiency was >95% as assessed by immunocytochemistry in all cases. Where indicated, cells were treated with MLi2 (MRC PPU, Dundee, UK), or with the equivalent volume of DMSO before fixation.

### Immunocytochemistry

HEK293T and A549 cells were fixed with 4% paraformaldehye (PFA) in PBS for 15 min at room temperature, followed by permeabilization with 0.2% Triton-X100/PBS for 10 min at room temperature. Coverslips were incubated in blocking solution [0.5% BSA (w/v) in 0.2% Triton-X100/PBS] for 1 h at room temperature and incubated with primary antibodies in blocking solution overnight at 4°C. Primary antibodies included rabbit polyclonal anti-pericentrin (Abcam, ab4448, 1:1000), mouse monoclonal anti-flag (Sigma-Aldrich, clone M2, F1804, 1:500), rat monoclonal anti-HA (Sigma-Aldrich, 11867423001, 1:500), mouse monoclonal anti-HA (Sigma-Aldrich, H3663, 1:1000), rabbit monoclonal anti-LRRK2 (UDD3; Abcam, ab133518, 1:1000), rabbit polyclonal anti-ACDB3 (Sigma-Aldrich, HPA015594, 1:500), mouse monoclonal anti-EEA1 (BD Biosciencs, 610457, 1:250), rabbit polyclonal anti-transferrin receptor (ThermoFisher, PA5-27739, 1:100), mouse monoclonal anti-Rab11 (BD Bioscinces, 610656, 1:100), mouse monoclonal anti-LAMP2 (Santa Cruz, sc-18822, 1:50), mouse monoclonal anti-γ-tubulin (Abcam, ab11316, 1:1000), rabbit polyclonal anti-RILPL1 (Sigma-Aldrich, HPA-041314, 1:300), sheep polyclonal anti-RILPL1 (1:50), rabbit monoclonal knockout-validated anti-pT73-Rab10 (Abcam, ab241060, 1:1000), or rabbit polyclonal anti-pT72-Rab8a (1:500, generous gift from Dario Alessi).

For centrosome staining of A549 cells, cells were fixed with 4% paraformaldehye (PFA) in PBS for 15 min at room temperature, followed by MeOH (stored at −20°C) for 5 min at 4°C. For staining of A549 cells with phospho-Rab10 antibody, cell permeabilization and blocking was performed simultaneously using 0.1% saponin in PBS containing 1% BSA (w/v) and 1% (v/v) goat serum (Vector Laboratories) (blocking solution) for 30 min at room temperature, and antibody incubations performed in blocking solution overnight at 4°C.

For immunocytochemistry of MEFs, cells were fixed with ice-cold MeOH at −20°C for 10 min, followed by permeabilization in ice-cold 0.1% Triton-X100/PBS for 5 min, and coverslips were blocked with 1% BSA in 0.1% Triton-X100/PBS for 1 h at room temperature. For determination of phospho-Rab10 colocalization with centrosome staining in MEFs, cells were fixed with 4% paraformaldehyde (PFA) in PBS for 15 min at 37°C, followed by ice-cold MeOH for 10 min at −20°C. Cells were permabilized in 0.5% Triton-X100/PBS for 15 min, followed by blocking in 0.5% BSA in 0.5% Triton-X100/PBS for 1 h at room temperature. For determination of ciliogenesis in MEFs, cells at around 70-80% confluency were grown for 12 h in the absence of serum in either the presence of DMSO or MLi2 (200 nM). Cells were fixed in 4% paraformaldehyde (PFA) in PBS for 15 min at 37°C, followed by permeabilization in 0.5% Triton-X100/PBS for 15 min at room temperature. Coverslips were blocked with 0.5% BSA in 0.5% Triton-X100/PBS for 1 h at room temperature and incubated with primary antibodies overnight at 4°C. Primary antibodies included rabbit polyclonal anti-Arl13b (Proteintech, 177-11-1-AP, 1:200) and mouse monoclonal anti-polyglutamylated tubulin (AdipogenSciences, AG-20B-0020-C100, 1:1000).

Immunocytochemistry employing sheep antibodies was performed sequentially, with the sheep antibody employed first. Upon overnight incubation with antibodies, coverslips were washed twice with 0.2% Triton-X100/PBS or 0.1% saponin/PBS (wash buffer), followed by incubation with secondary antibodies in wash buffer for 1 h at room temperature. Secondary antibodies included Alexa405-conjugated goat anti-mouse, Alexa488-conjugated goat anti-mouse, goat anti-rabbit or donkey anti-sheep, Alexa555-conjugated goat-anti-mouse, goat anti-rabbit or donkey anti-sheep, and Alexa647-conjugated goat anti-mouse, goat anti-rabbit or donkey anti-sheep (all from Invitrogen, 1:1000). Coverslips were washed twice in wash buffer, rinsed in PBS and mounted in mounting medium with DAPI (Vector Laboratories).

### Cell-polarization assays

For wound healing assays to determine cell polarization, MEF cells were grown to confluency on glass coverslips in a 24-well plate. Cells were incubated for 12 h with 200 nM MLi2 or DMSO before the scratch. The following day, a scratch wound was generated down the middle of the coverslip with a p10 pipette tip, cells were gently washed twice in full medium, followed by addition of full medium containing 200 nM MLi2 or DMSO and incubation for various times before fixation. Cells were fixed with 4% PFA for 20 min at room temperature, followed by permeabilization in 0.5% Triton-X100/PBS for 15 min, and blocking with 0.5% BSA in 0.5% Triton-X100/PBS for 1 h at room temperature. Coverslips were processed for immunocytochemistry as described above in 0.5% Triton-X100/PBS containing 0.5% BSA (w/v) and employing a rabbit polyclonal anti-ACBD3 antibody (Sigma-Aldrich, HPA015594, 1:500), and images were acquired on an Olympus FV1000 Fluoview confocal microscope using a 20x objective lens. For determination of cell polarity, the first row of cells facing the wound was analyzed, with 100-150 cells scored for each condition and experiment. Cells were scored as polarized when the Golgi was located in a 120° sector emerging from the center of the nucleus and facing the wound edge (Etienne-Manneville and Hall, 2001). Basal levels of expected random orientation of 33% were confirmed by analyzing Golgi orientation immediately after generating the wound.

### Image acquisition and quantification

Images were acquired on a Leica TCS-SP5 confocal microscope using a 63×1.4 NA oil UV objective (HCX PLAPO CS) (Lara Ordóñez et al., 2019), or on an Olympus FV1000 Fluoview confocal microscope using a 60×1.2 NA water objective lens. Images were collected using single excitation for each wavelength separately and dependent on secondary antibodies, and the same laser intensity settings and exposure times were used for image acquisition of individual experiments to be quantified. Around 10-15 optical sections of selected areas were acquired with a step size of 0.5 µm, and maximum intensity projections of z-stack images analyzed and processed using Leica Applied Systems (LAS AF6000) image acquisition software or ImageJ. Since the average distance between duplicated centrosomes is cell-type-dependent, we quantified the distances between duplicated centrosomes from around 100 non-transfected cells, with mitotic cells excluded from the analysis in all cases. In U2OS cells, around 90-95% of cells display a distance between duplicated centrosomes <2 µm, and centrosome splitting in this cell type has been defined when the duplicated centrosomes are >2 µm apart (Meraldi and Nigg, 2001). Performing identical analyses by measuring the distances between duplicated centrosomes from around 100 non-transfected wildtype cells, centrosomes were scored as being split when the distance between their centers was >1.5 µm for HEK293T cells, >2.5 µm for A549 cells, or >5 µm for MEF cells, respectively (Madero-Pérez et al., 2018; Lara Ordóñez et al., 2019; Fdez et al., 2022). For each condition and experiment, distances were scored from around 30 transfected cells with duplicated centrosomes (HEK293T and A549 cells) or from around 50 cells (MEFs) with duplicated centrosomes, and mitotic cells were excluded from the analysis in all cases. Quantification of centrosomal distances was performed by an additional observer blind to condition, with identical results obtained in both cases. For quantification of Golgi morphology in MEF cells, the Golgi was considered split when two discrete Golgi stacks (usually on opposite sides of the nucleus) could be distinguished and fragmented when >3 distinct stacks (or a full dispersal into vesicles) was observed. For Golgi morphology determination, around 100-150 cells were scored per experiment and condition.

### Electron microscopy, sample preparation and imaging

A549 cells were cultured in glass-bottom MatTek dishes (MatTek Life Sciences, P35G-0-14-C) and transfected with RILPL1-miniSOG-HA using LipoD293™ Transfection Reagent (SignaGen Laboratories, SL100668) as described above. Proteins were allowed to express for 48 h and cells were processed as previously described (Boassa et al., 2013; Boassa et al., 2019). Briefly, cells were rinsed with pre-warmed HBSS and fixed using pre-warmed 2.5% (w/v) glutaraldehyde (Electron Microscopy Sciences, 16220), 0.1% tannic acid (w/v) (Electron Microscopy Sciences, 21700), 3 mM calcium chloride in 0.1 M sodium cacodylate buffer pH 7.4 (Ted Pella Incorporated, 18851) for 5 min at 37°C and then on ice for 1 h. Subsequent steps were performed on ice, cells were rinsed five times using chilled 0.1 M sodium cacodylate buffer pH 7.4 (wash buffer) and treated for 30 min in a blocking solution (50 mM glycine, 10 mM KCN, 20 mM aminotriazole and 0.01% hydrogen peroxide in 0.1 M sodium cacodylate buffer pH 7.4) to reduce non-specific background precipitation of DAB.

Cells were first imaged with minimum light exposure to identify transfected cells for correlative light and electron microscopy (CLEM) using a Leica SPE II inverted confocal microscope outfitted with a stage chilled to 4°C. For photo-oxidation, DAB (3-3′-diaminobenzidine, Sigma-Aldrich, D8001-10G) was dissolved in 0.1 N HCl at a concentration of 5.4 mg/ml and subsequently diluted 10-fold into blocking solution, mixed, and passed through a 0.22 µm syringe filter before use. DAB solution was freshly prepared prior to photo-oxidation and placed on ice protected from light. DAB solution was added to the MatTek dish and regions of interest were illuminated through a standard FITC filter set (EX470/40, DM510, BA520) with intense light from a 150 W Xenon lamp. Photo-oxidation was stopped as soon as an optically-dense brown reaction product began to appear in place of the miniSOG intrinsic green fluorescence signal, monitored by transmitted light (around 4-6 min). Multiple areas on a single MatTek dish were photo-oxidized.

Subsequently, plates were placed on ice and washed five times for 2 min each with ice-cold wash buffer to remove unpolymerized DAB. After washing out DAB, cells were post-fixed with 2% reduced osmium tetroxide (Electron Microscopy Sciences, 19190) (2% osmium tetroxide, 1.5% KFeCN in 0.1 M sodium cacodylate buffer pH 7.4) for 1 h on ice, then washed with ice-cold double-distilled water three times for 1 min. Some samples were additionally stained overnight with filtered 2% uranyl acetate (Electron Microscopy Sciences, 22400) in double-distilled water and compared to others in which this step was omitted. The following day, plates were washed three times for 1 min with double-distilled water and were dehydrated with an ice-cold graded ethanol series (20%, 50%, 70%, 90%, 100%, 100%, 3 min each) and washed once at room temperature anhydrous ethanol (3 min). Samples were then embedded in Durcupan™ ACM resin (Sigma-Aldrich; Durcupan™ ACM component A, M epoxy resin (44611); Durcupan™ ACM component B, hardener 964 (44612); Durcupan™ ACM component C, accelerator 960 (44613); Durcupan™ ACM component D (44614)) using a 1:1 mixture of anhydrous ethanol:Durcupan™ ACM resin for 30 min on a platform with gentle rocking, followed by incubation with 100% Durcupan™ ACM resin overnight with rocking. The following day, the resin was removed from MatTek dishes by decanting and gentle scraping without touching the cells and changed with freshly prepared resin for 1 h three times. After third replacement, resin was polymerized in a vacuum oven at 60°C for 48 h under 10 mm Hg vacuum pressure atmosphere.

Photo-oxidized areas of interest were identified by transmitted light, sawed out using a jeweller's saw, and mounted on dummy acrylic blocks with cyanoacrylic adhesive. The coverslip was carefully removed, the resin was trimmed, and ultrathin sections (80 nm thick) were cut using a diamond knife (Diatome). Electron micrographs were recorded using a FEI Tecnai™ 12 Spirit transmission electron microscope (TEM) operated at 80 kV. For electron tomography, thicker sections (750 nm) were imaged on a FEI Titan Halo™ microscope operated at 300 kV in scanning mode, the scattered electrons being collected on a high-angle annular dark-field detector. Prior to imaging, the luxel grids carrying the specimen serial sections were coated with carbon on both sides; colloidal gold particles (10, 20 and 50 nm diameter) were deposited on each side of the sections to serve as fiducial markers. Because centrosomes cannot be clearly distinguished on electron micrographs of thick sections, a preliminary tomography run was first implemented using a low magnification setting on the cells of interest (spanning a 12 µm×12 µm area). This allowed for identification of the exact sections containing centrosomal areas; higher resolution tomograms (with a ∼1 nm pixel size) were then acquired on the spot. For each tomogram, four tilt series were collected using the SerialEM package. For each series, the sample was tilted from −60 to +60°, every 0.5°. Tomograms were generated using an iterative reconstruction procedure (Phan et al., 2017).

### Western blotting

HEK293T and A549 cells were collected 48 h after transfection (MEF cells at 80% confluency) from a well of a six-well plate. Cells were washed in PBS, and the cell pellet was resuspended in 75 µl PBS. Cells were lysed with 25 µl of 4× Nu-PAGE LDS sample buffer (Novex, Life Technologies, NP00008) supplemented with β-mercaptoethanol to a final volume of 2.5% (v/v), sonicated and boiled at 70°C for 10 min. Around 10-15 µl (around 20 µg of protein) were resolved by SDS-PAGE polyacrylamide gel electrophoresis using 4-20% precast gradient gels (Bio-Rad, 456-1096), and proteins were electrophoretically transfected onto nitrocellulose membranes (GE Healthcare). Membranes were blocked in blocking buffer (Li-COR Biosciences, Li-COR Odyssey intercept blocking buffer, 927-70001) for 1 h at room temperature, and incubated with primary antibodies in blocking buffer overnight at 4°C. Primary antibodies included mouse monoclonal anti-GFP (Sigma-Aldrich, 11814460001, 1:1000), mouse monoclonal anti-flag (Sigma-Aldrich, clone M2, F1894, 1:500), rat monoclonal anti-HA (Sigma-Aldrich, 118674123001, 1:500), mouse monoclonal anti-Rab8 (BD, 610844, 1:500), mouse monoclonal knockout-validated anti-Rab10 (Sigma-Aldrich, SAB5300028, 1:1000), rabbit monoclonal anti-pT72-Rab8a (Abcam, ab230260, 1:1000), rabbit monoclonal anti-pT73-Rab10 (Abcam, ab230261, 1:1000), rabbit monoclonal anti-S935-LRRK2 (Abcam, ab133450, 1:500), mouse monoclonal anti-LRRK2 (AntibodiesInc, 75-253 (N241A/34), 1:1000), mouse monoclonal anti-α-tubulin (Sigma-Aldrich, clone DM1A, T6199, 1:25,000) and mouse monoclonal anti-GAPDH (Santa Cruz, sc-32233, 1:2000). Sheep polyclonal antibodies against Rab12, Rab35 and Rab43 (generous gifts from Dario Alessi) were employed at 1:100 dilution in 5% milk in 0.1% Tween-20/TBS blocking buffer overnight at 4°C, and membranes were developed using ECL Prime Western Blotting Detection Reagent (GE Healthcare) as previously described (Lara Ordóñez et al., 2019).

### Statistical analysis

One-way ANOVA with Tukey's *post hoc* test was employed, with significance set at *P*<0.05. Significance values for all data are indicated in the figure legends. All statistical analysis and graphs were performed with Prism software version 7.0 (GraphPad, San Diego, CA, USA).

## Supplementary Material

Supplementary information
