# Supplementary material for: The LRRK2 signaling network converges on a centriolar phospho-Rab10/RILPL1 complex to cause deficits in centrosome cohesion and cell polarization
Source: Biol Open. 2022 Jul 29;11(8):bio059468. doi: 10.1242/bio.059468 (PMC9346292; doi:10.1242/bio.059468)
Supplement: Supplementary information [file biolopen-11-059468-s1.pdf]

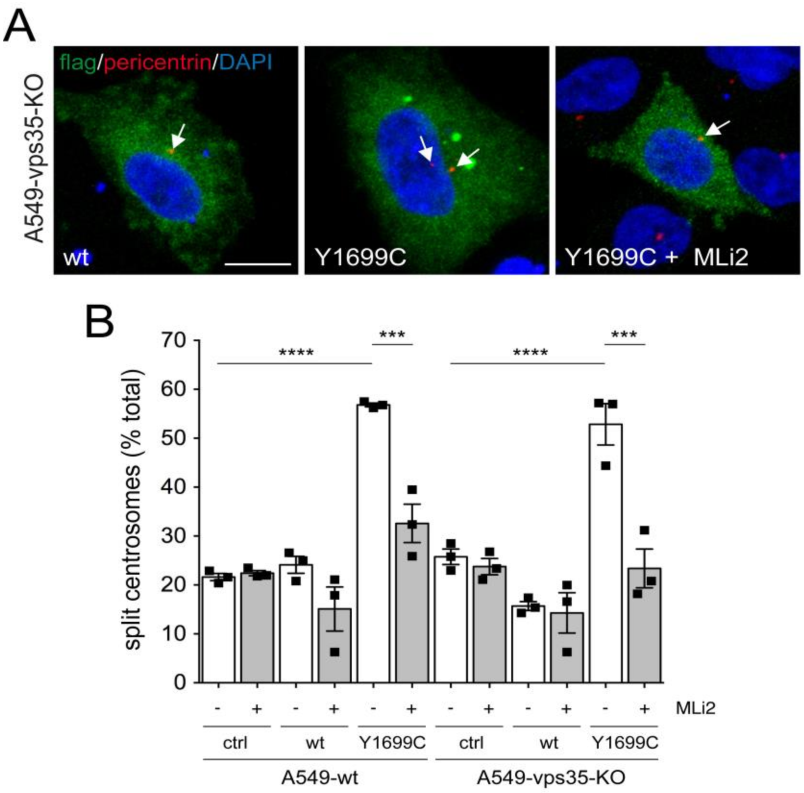

Fig. S1. Pathogenic LRRK2-mediated centrosomal cohesion deficits in A549 wildtype and vps35-KO cells. (**A**) Example of A549 vps35-KO cells transfected with either flag-tagged wildtype (wt) or Y1699C-mutant LRRK2, and either treated or untreated with 200 nM MLi2 for 2 h prior to immunocytochemistry with antibodies against flag, pericentrin and with DAPI. Arrows point to centrosomes in transfected cells. Scale bar, 10 µm. (**B**) Quantification of the percentage of A549-wt or A549-vps35-KO cells with duplicated centrosomes > 2.5 µm apart (split centrosomes) either in the absence of transfection (ctrl), or upon transfection with wt or Y1699C-mutant LRRK2. Cells were treated with or without MLi2 (200 nM, 2 h) prior to immunocytochemistry as indicated. Bars represent mean ± S.E.M. (n=3 independent experiments); ****p < 0.001; ***p < 0.005.

Biology Open • Supplementary information

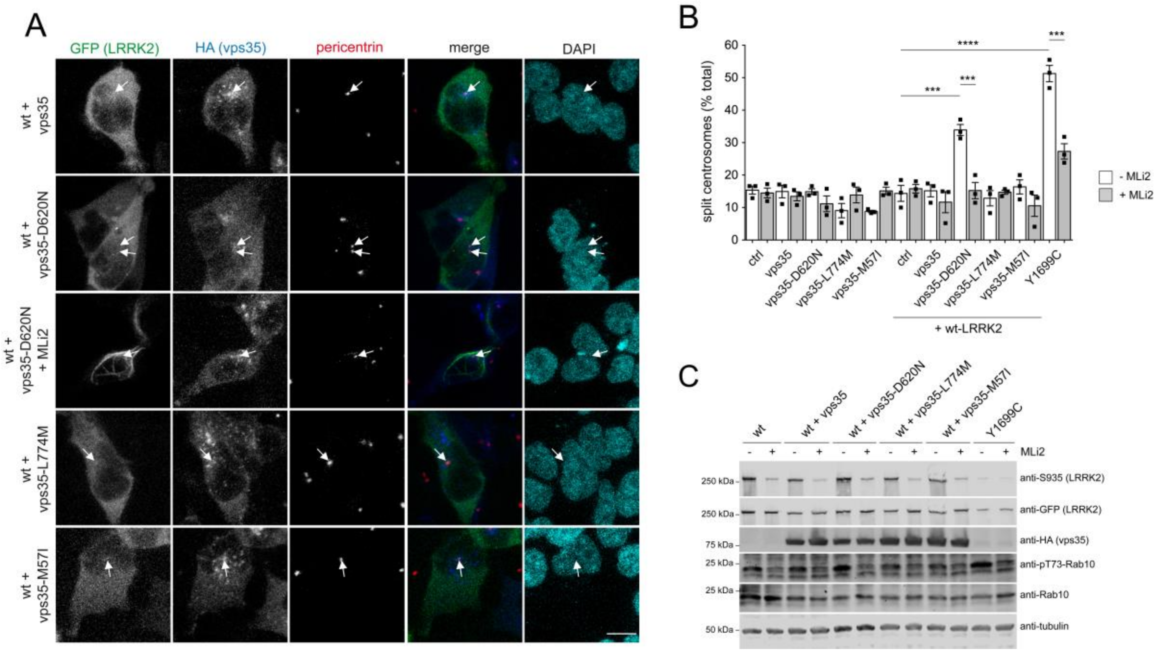

Fig. S2. Co-expression of vps35-D620N with wildtype LRRK2 causes centrosomal cohesion deficits dependent on the LRRK2 kinase activity. (**A**) HEK293T cells transfected with GFP-tagged wildtype LRRK2 (wt) and HA-tagged wildtype or mutant vps35 constructs, and treated with or without MLi2 (100 nM, 2 h) prior to immunocytochemistry as indicated. Cells were stained with an antibody against the HA-tag (Alexa-647 secondary antibody; pseudocolored in blue), an antibody against pericentrin (Alexa-555 secondary antibody; red) and DAPI (cyan). Arrows point to centrosomes in transfected cells. Note that whilst the vps35-D620N mutation is pathogenic, L774M and M57I are rare variants of unknown significance. Scale bar, 10 μ m. (**B**) Quantification of the percentage of cells with duplicated split centrosomes transfected with pCMV (ctrl) or different HA-tagged vps35 constructs, or co-transfected with GFP-tagged wildtype LRRK2, in the absence or presence of MLi2 (100 nM, 2 h) as indicated. Bars represent mean ± S.E.M. (n=3 experiments); ****p < 0.001; ***p < 0.005. (**C**) Cells were transfected with the indicated constructs, left untreated or treated with MLi2 (100 nM, 2 h) as indicated, and extracts blotted for GFP-tagged LRRK2, phosphorylated LRRK2 (S935), phosphorylated Rab10 (pT73-Rab10), total Rab10, and tubulin as loading control.

Biology Open • Supplementary information

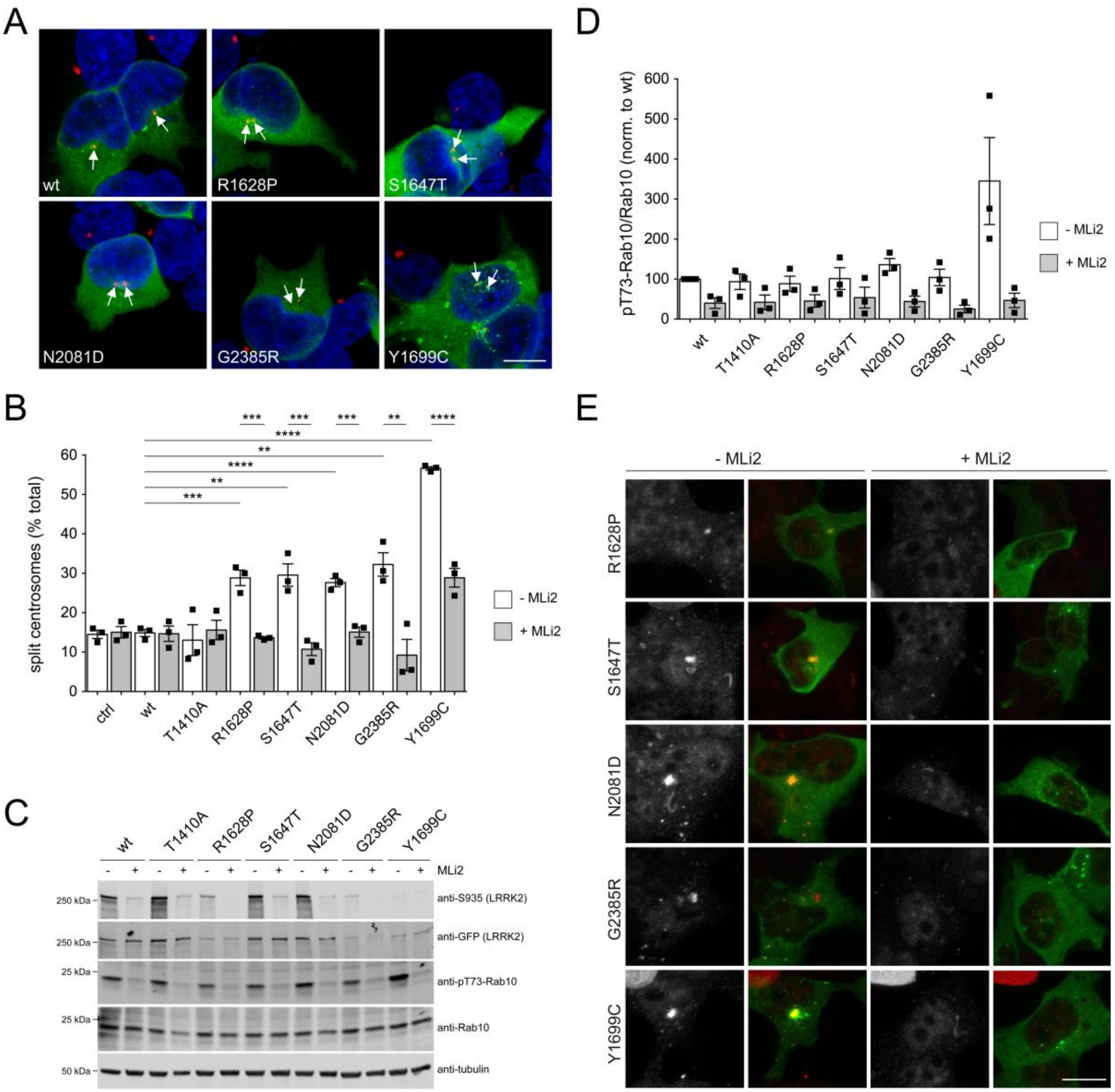

Fig. S3. LRRK2 risk variants cause centrosomal cohesion deficits in HEK293T cells. (**A**) HEK293T cells were transfected with GFP-tagged wildtype (wt) LRRK2, a point mutant described to be non-pathogenic (T1410A), LRRK2 risk variants or pathogenic Y1699C LRRK2 as indicated. Cells were stained for the centrosomal marker pericentrin (red) and DAPI (blue). Arrows point to centrosomes in transfected cells. Scale bar, 10 μm. (**B**) Quantification of the percentage of cells with duplicated split centrosomes from either non-transfected (ctrl) cells, or from cells transfected with the indicated constructs in the absence or presence of MLi2 (100 nM, 2 h) as indicated. Bars represent mean ± S.E.M. (n=3 experiments); ****$p < 0.001$; ***$p < 0.005$; **$p < 0.01$. (**C**) Cells were

Biology Open • Supplementary information

transfected with the indicated constructs, left untreated or treated with MLi2 (100 nM, 2 h), and extracts blotted for GFP-tagged LRRK2, phosphorylated LRRK2 (S935), phosphorylated Rab10 (pT73-Rab10), total Rab10, and tubulin as loading control. (**D**) Quantification of endogenous phospho-Rab10/Rab10 levels (normalized to values from cells transfected with wt LRRK2) from experiments depicted in (C). Bars represent mean ± S.E.M. (n=3 independent experiments). Only the pathogenic Y1699C-LRRK2 mutant construct displays a pronounced increase in phospho-Rab10/Rab10 levels. (**E**) Cells were transfected with the indicated GFP-tagged constructs, left untreated or treated with MLi2 as indicated, and stained with an antibody against endogenous phospho-Rab10 (red). Scale bar, 10 μm.

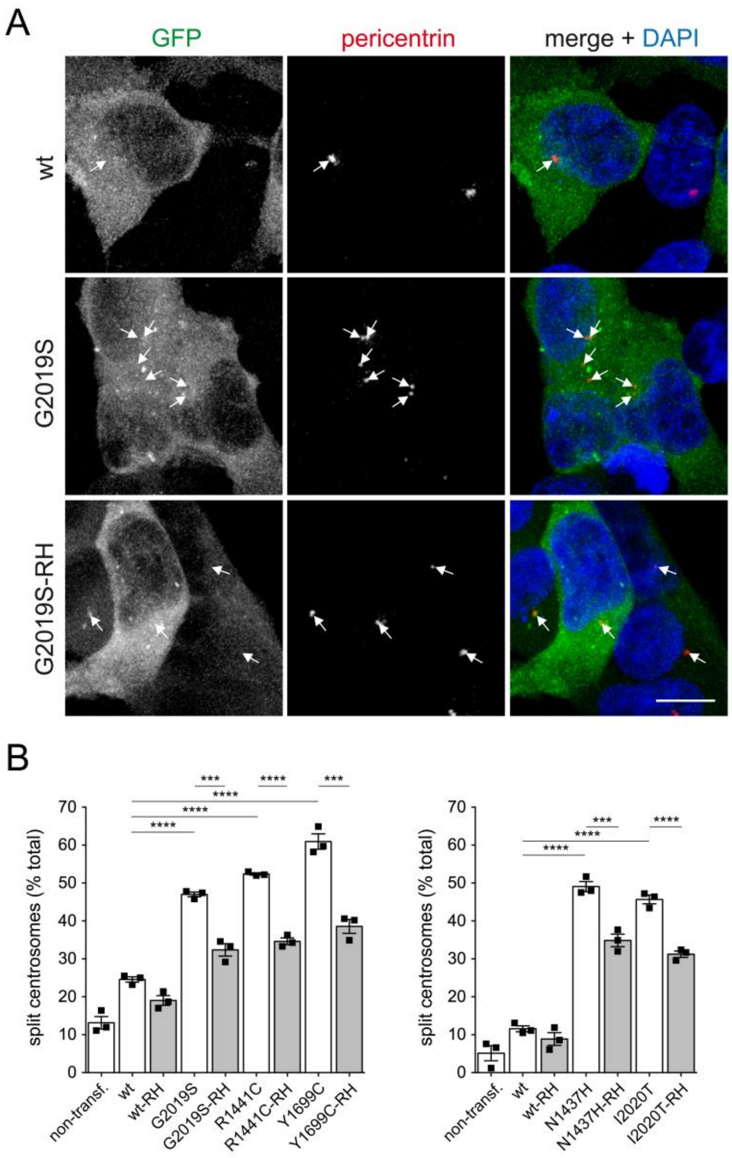

Fig. S4. The protective R1398H variant decreases centrosomal cohesion deficits mediated by pathogenic LRRK2. (**A**) HEK293T cells were transfected with GFP-tagged wildtype (wt) LRRK2, pathogenic G2019S mutant, or pathogenic G2019S mutant containing the protective R1398H variant (G2019S-RH). Cells were stained for the centrosomal marker pericentrin (red) and DAPI (blue). Arrows point to centrosomes in transfected cells. Scale bar, 10 μm. (**B**) Quantification of the percentage of cells with split centrosomes from either non-transfected cells, or from cells transfected with the indicated constructs. Bars represent mean ± S.E.M. (n=3 experiments); ****p < 0.001; ***p < 0.005.

Biology Open • Supplementary information

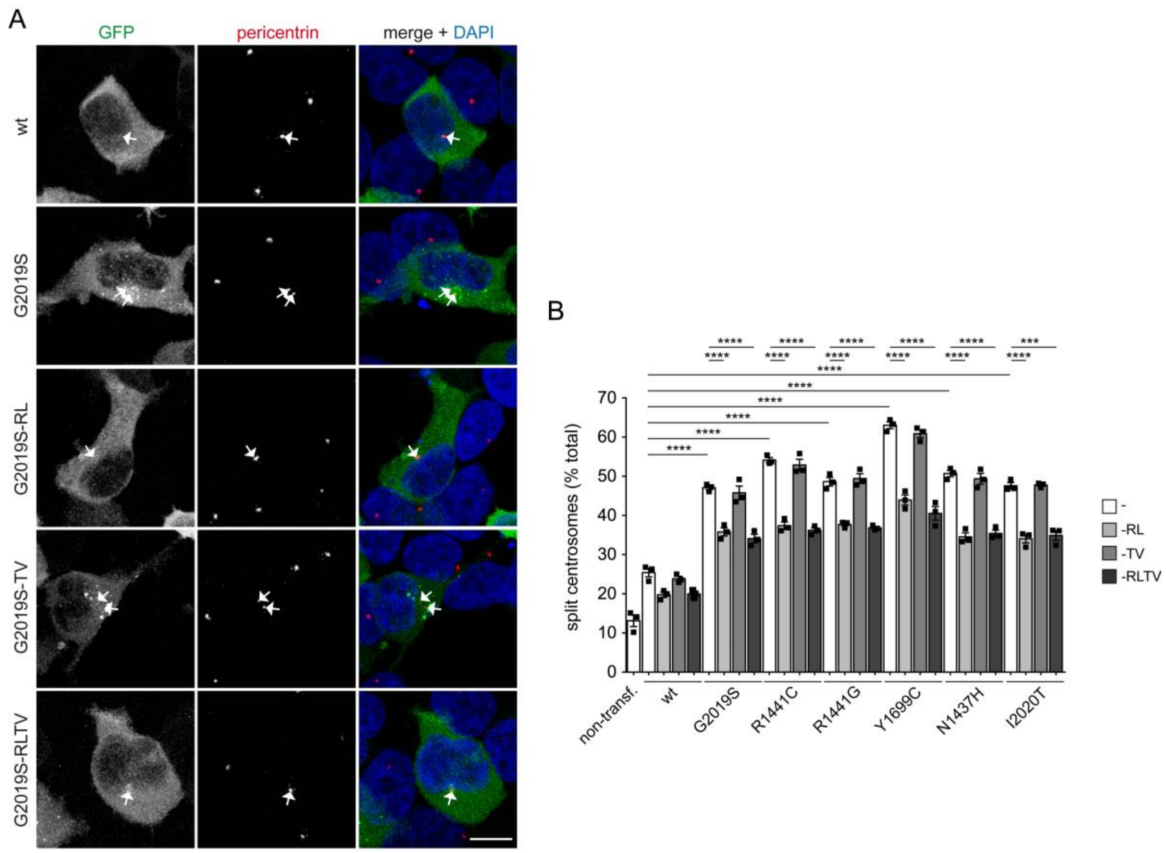

Fig. S5. Synthetic variants which modulate GTP binding/hydrolysis decrease centrosomal cohesion deficits mediated by pathogenic LRRK2. (**A**) HEK293T cells were transfected with GFP-tagged wildtype (wt) LRRK2, pathogenic G2019S mutant, pathogenic G2019S mutant containing the synthetic R1398L variant (G2019S-RL), the T1343V mutation (G2019S-TV), or both (G2019S-RLTV). Cells were stained for the centrosomal marker pericentrin (red) and DAPI (blue). Arrows point to centrosomes in transfected cells. Scale bar, 10 μm. (**B**) Quantification of the percentage of cells with duplicated split centrosomes from either non-transfected cells or from cells transfected with the indicated constructs. Bars represent mean ± S.E.M. (n=3 experiments); ****$p < 0.001$; ***$p < 0.005$.

Biology Open • Supplementary information

Biology Open (2022): doi:10.1242/bio.059468: Supplementary information

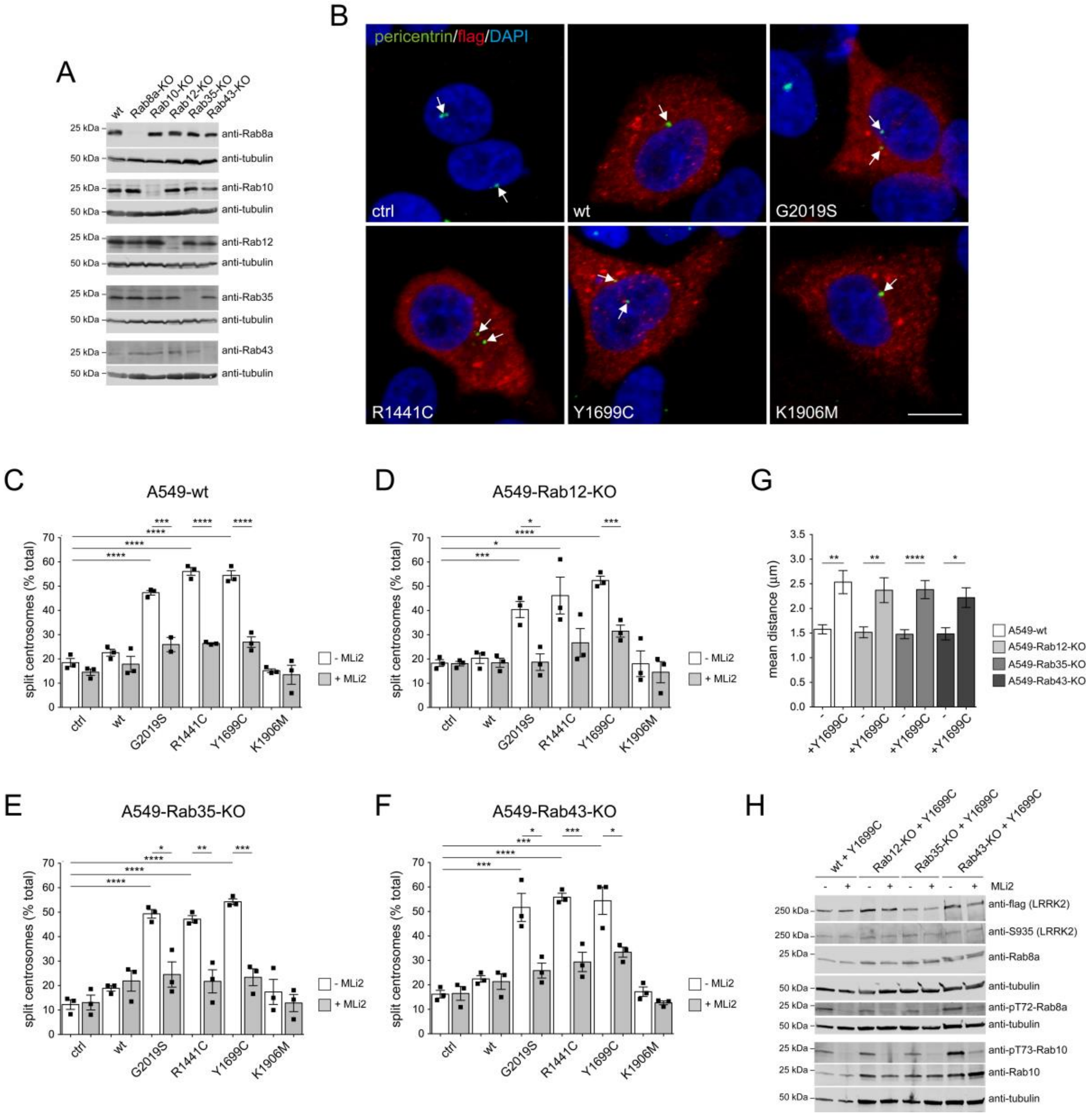

Fig. S6. Pathogenic LRRK2-mediated centrosomal cohesion deficits are independent of Rab12, Rab35 or Rab43. (**A**) Wildtype A549 cells (wt), or cells where the distinct Rab proteins had been knocked out (KO) using CRISPR-Cas9 were subjected to immunoblot analysis for the presence or absence of the various Rab proteins as indicated, with tubulin as loading control. (**B**) Example of A549 Rab12-KO cells transfected with either pCMV (ctrl), or with the indicated flag-tagged LRRK2 constructs and stained with antibodies against flag, pericentrin and with DAPI. Scale bar, 10 µm. (**C**) Quantification of the

percentage of wildtype A549 cells with duplicated split centrosomes (duplicated centrosomes with a distance between their centres > 2.5 μm) transfected with the different LRRK2 constructs, and either left untreated or incubated with 200 nM MLi2 for 2 h prior to immunocytochemistry as indicated. (**D**) Same as in (C), but employing Rab12-KO cells. (**E**) Same as in (C), but employing Rab35-KO cells. (**F**) Same as in (C), but employing Rab43-KO cells. In all cases, bars represent mean ± S.E.M. (n=3 experiments); ****p < 0.001; ***p < 0.005; **p < 0.01; *p < 0.05. (**G**) Wildtype cells, or Rab12-KO, Rab35-KO or Rab43-KO cells were transfected with flag-tagged Y1699C LRRK2, and distances between duplicated centrosomes quantified from around 50-70 transfected or non-transfected cells each. ****p < 0.001; **p < 0.01; *p < 0.05. (**H**) A549 wt cells, or Rab12-KO, Rab35-KO or Rab43-KO cells were transfected with Y1699C-mutant LRRK2 construct, left untreated or incubated with 200 nM MLi2 for 2 h as indicated, and extracts were blotted for flag-tagged LRRK2, phosphorylated LRRK2 (S935), pT73-Rab10, total Rab10, pT72-Rab8a, total Rab8a or tubulin as loading control.

Biology Open • Supplementary information

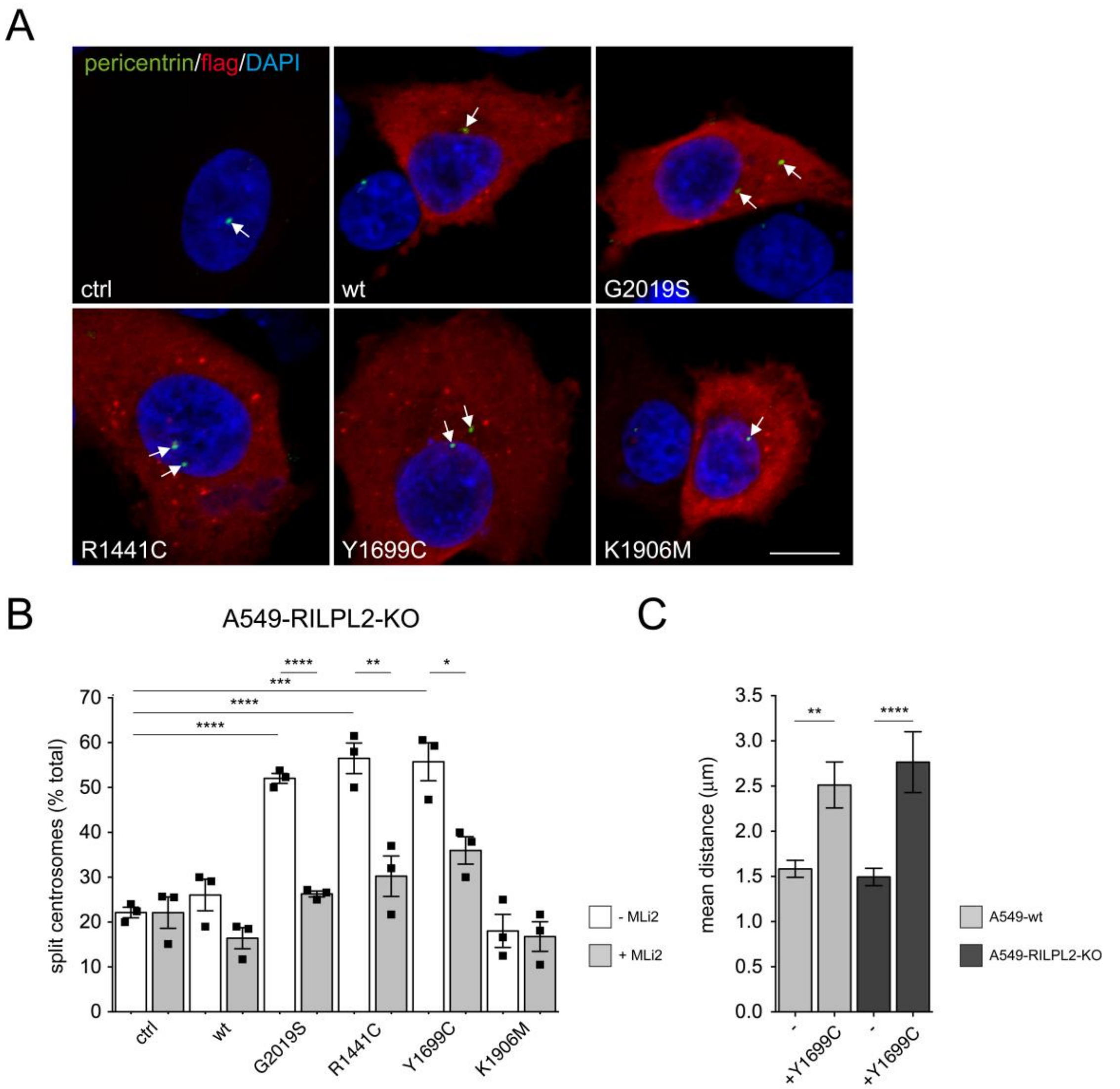

Fig. S7. RILPL2 is dispensable for the LRRK2-mediated centrosomal cohesion deficits. (**A**) RILPL2-KO cells were transfected with either pCMV (ctrl), or with the indicated flag-tagged LRRK2 constructs, and stained with antibodies against flag, pericentrin and with DAPI. Scale bar, 10 µm. (**B**) Quantification of the percentage of A549 RILPL2-KO cells with duplicated split centrosomes upon transfection of the indicated constructs, in either the absence or presence of MLi2 (200 nM, 2 h) prior to immunocytochemistry. Bars represent mean ± S.E.M. (n=3 experiments); ****p < 0.001; ***p < 0.005; **p < 0.01; *p < 0.05. (**C**) Wildtype or RILPL2-KO cells were transfected with flag-tagged Y1699C LRRK2, and distances between duplicated centrosomes quantified from around 50-70 transfected or non-transfected cells each. ****p < 0.001; **p < 0.01.

Biology Open • Supplementary information

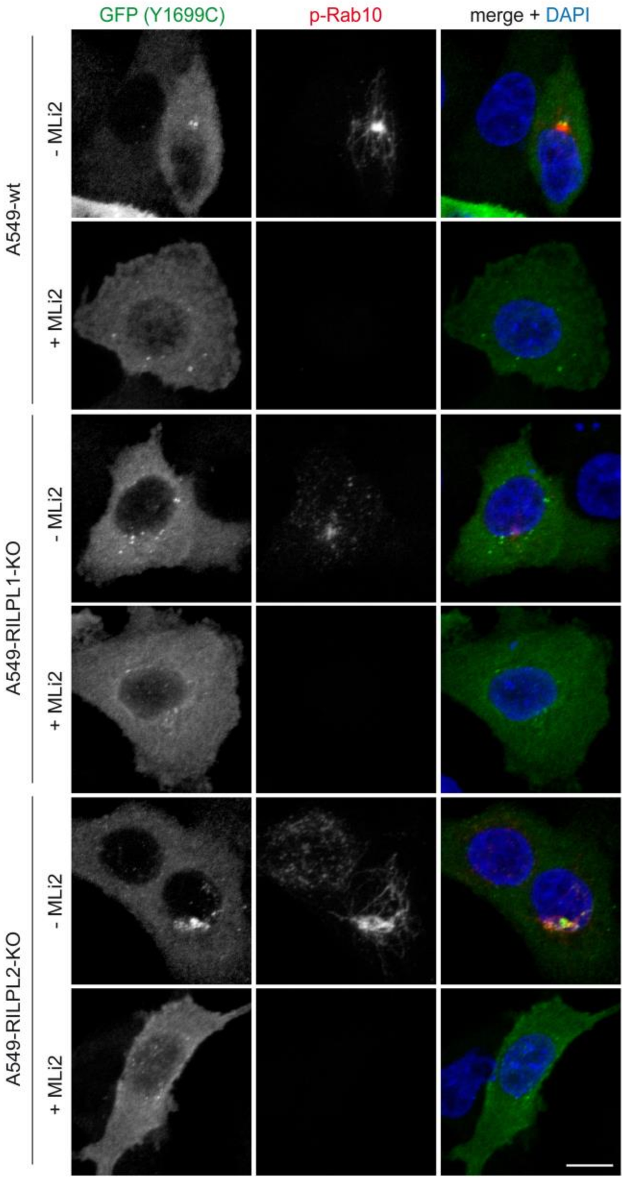

Fig. S8. Localization of phospho-Rab10 is influenced by the presence of RILPL1 but not RILPL2. Examples of wildtype, RILPL1-KO or RILPL2-KO A549 cells transfected with flag-tagged Y1699C LRRK2, and treated with or without MLi2 (200 nM, 2 h) before immunostaining with an antibody against phospho-Rab10 (p-Rab10) and with DAPI. Perinuclear phospho-Rab10 clusters are prominent in A549 wildtype cells expressing pathogenic LRRK2. In RILPL1-KO cells, perinuclear clusters are rarely observed, but phospho-Rab10 displays an additional punctate staining throughout the cytosol. In RILPL2-KO cells, perinuclear phospho-Rab10 staining is prominent in some cells expressing pathogenic LRRK2. Scale bar, 10 μm.

Biology Open • Supplementary information

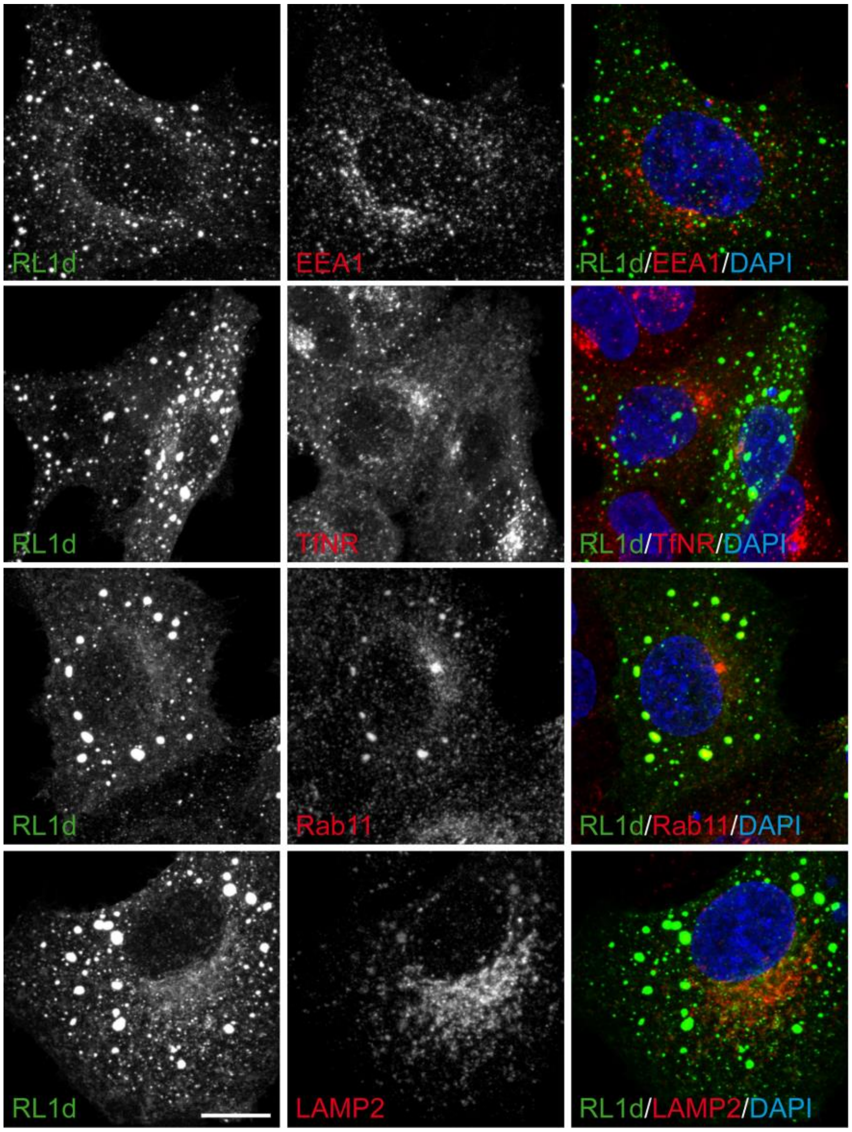

Fig. S9. C-terminal RILPL1-positive structures are negative for early endosomal or lysosomal markers. Example of A549 cells transfected with GFP-tagged C-terminal region of RILPL1 (RL1d) and stained with antibodies against the early endosome marker EEA1, the recycling endosome markers transferrin receptor (TfNR) or Rab11, or the lysosomal marker LAMP2 and with DAPI. Only Rab11 partially colocalizes with RL1d. Scale bar, 10 μm.

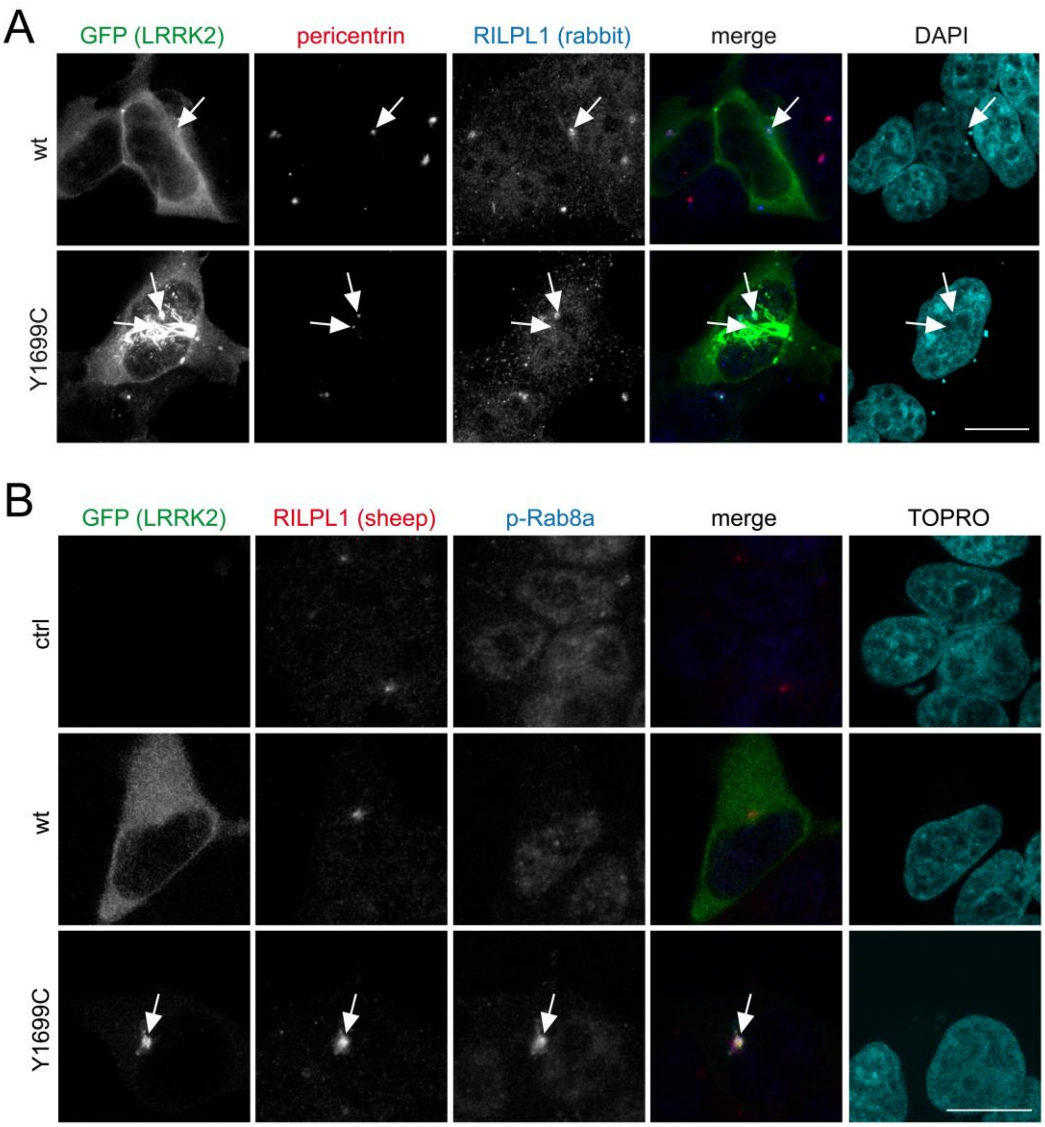

Fig. S10. Endogenous RILPL1 localizes to the centrosome and recruits phospho-Rabs in cells transfected with pathogenic LRRK2. (**A**) Example of HEK293T cells transfected with GFP-tagged wildtype or pathogenic Y1699C-LRRK2 and stained with antibodies against pericentrin (Alexa-647 secondary antibody, red), RILPL1 (Alexa-594 secondary antibody, pseudocolored in blue) and with DAPI (cyan). Arrows point to centrosomes in transfected cells. In the pathogenic LRRK2-expressing cell, endogenous RILPL1 colocalizes with only one of the duplicated centrosomes (note that upon centrosome duplication, only the "older" mother centriole contains subdistal appendages). Scale bar, 10 µm. (**B**) Example of non-transfected cells (ctrl), or cells transfected with GFP-tagged

Biology Open • Supplementary information

wildtype or Y1699C-LRRK2 and stained with antibodies against RILPL1 (Alexa-594 secondary antibody, red), phospho-Rab8 (Alexa-405 secondary antibody, pseudocolored in blue) and with TOPRO (cyan). Note that the phospho-Rab8 antibody detects both phospho-Rab8 and phospho-Rab10 by immunocytochemistry (10). Arrow points to endogenous phospho-Rab accumulation which colocalizes with endogenous RILPL1 in pathogenic LRRK2-expressing cell. Scale bar, 10 μm.

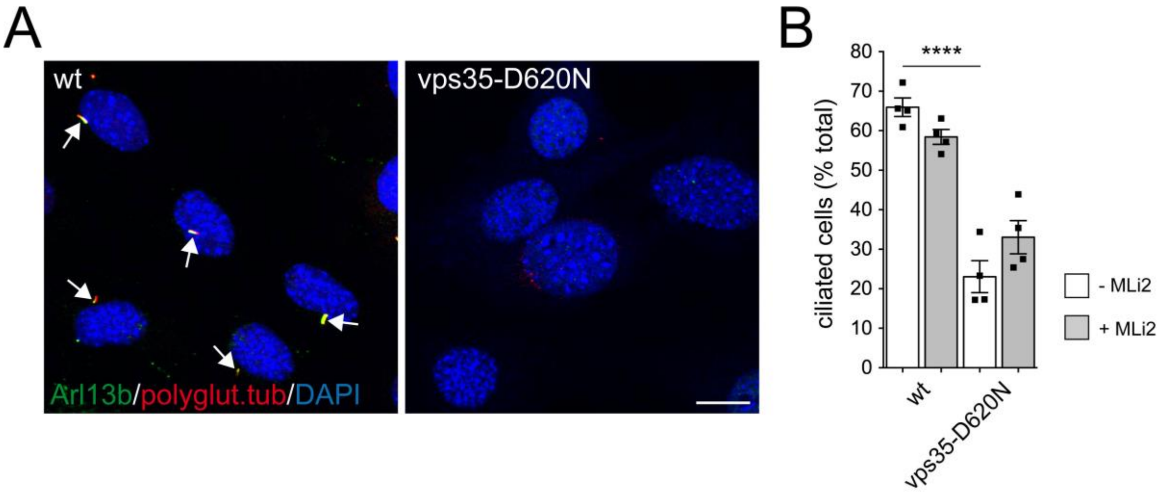

Fig. S11. Vps35-D620N MEF cells display ciliogenesis deficits. (**A**) Representative images of vps35-D620N and corresponding wildtype (wt) littermate MEFs stained with antibodies against two ciliary markers (Arl13b and polyglutamylated tubulin) and with DAPI. Arrows point to cilia. Scale bar, 10 µm. (**B**) Ciliogenesis quantification in wt and vps35-D620N MEFs in either the absence or presence of MLi2 (200 nM, 12 h). Bars represent mean ± S.E.M. (n=4 independent experiments); ****$p < 0.001$. Treatment with MLi2 only evoked a slight, non-significant reversal of the ciliogenesis deficit in the vps35-D620N MEFs. In all cases, the presence of cilia was quantified from around 200 cells per condition and experiment.

Biology Open • Supplementary information

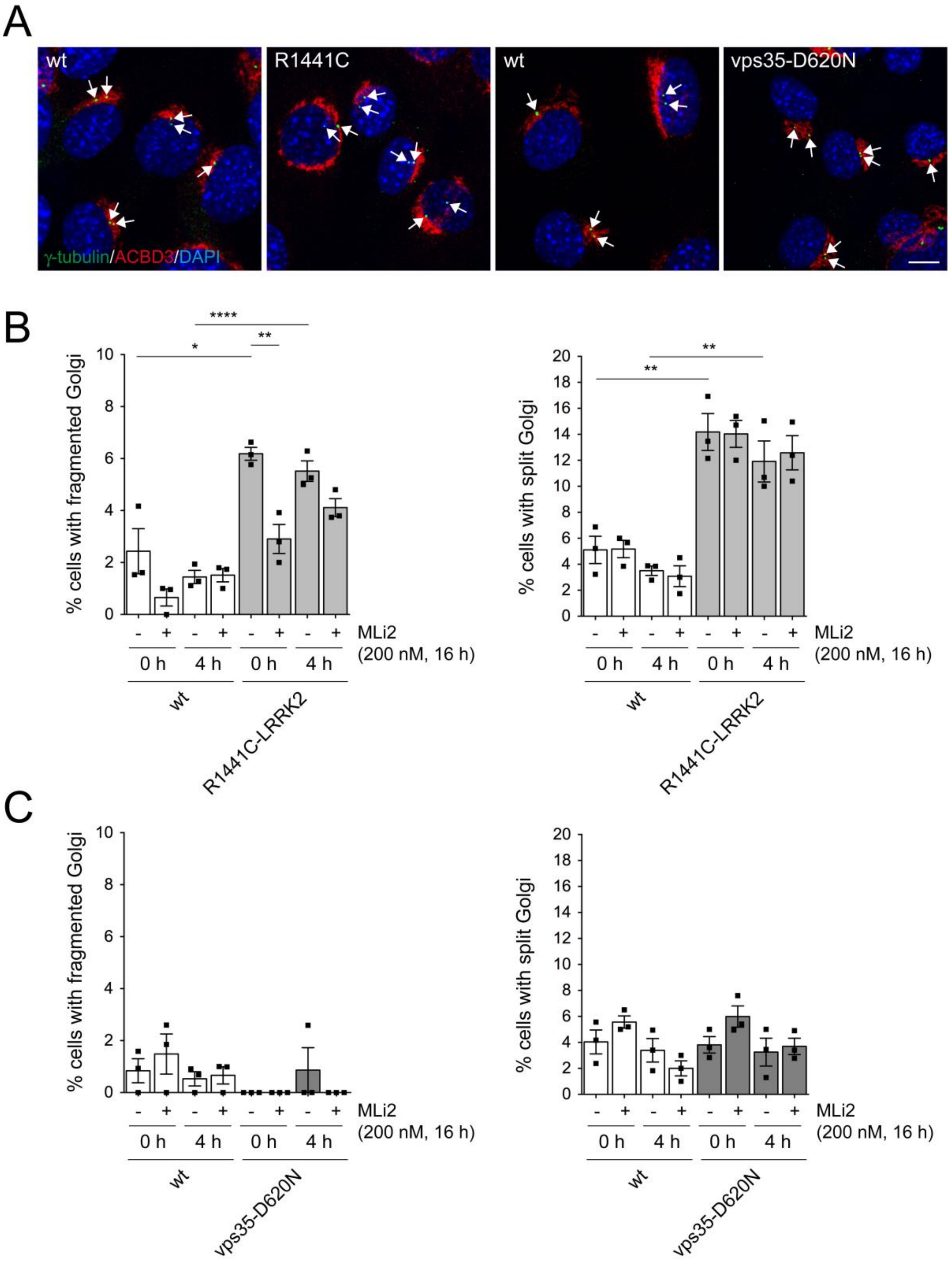

Fig. S12. Altered Golgi morphology in R1441C-LRRK2 but not vps35-D620N MEF cells. (**A**) Representative images of wildtype (wt) littermate and R1441C-LRRK2 MEFs, and respective wt littermate and vps35-D620N MEFs stained with antibodies against a

centrosomal marker (γ-tubulin), a Golgi marker (ACBD3) and DAPI. Arrows point to centrosomes. Scale bar, 10 µm. (**B**) Quantification of Golgi morphology in wt littermate and R1441C-LRRK2 MEFs in either the absence or presence of MLi2 (200 nM, 16 h) and either at t = 0 h or t = 4 h after generating the scratch wound. Percentage of cells in the first row of the scratch wound displaying a fragmented Golgi (left; > 3 distinct stacks) or a split Golgi (right; two discrete Golgi stacks). N > 100 cells in the first row of cells facing the scratch were quantified for each condition and genotype. Bars represent mean ± S.E.M. (n=3 independent experiments); ****p < 0.001; **p < 0.01; *p < 0.05. (**C**) Same as in (B), but quantification from wt littermate and vps35-D620N MEF cells. Bars represent mean ± S.E.M. (n=3 independent experiments).
